# Imaging and outcome correlates of ctDNA methylation markers in prostate cancer: a comparative, cross-sectional [⁶⁸Ga]Ga-PSMA-11 PET/CT study

**DOI:** 10.1186/s13148-025-01811-5

**Published:** 2025-02-25

**Authors:** Kilian Kluge, Vincent Lotz, Holger Einspieler, David Haberl, Clemens Spielvogel, Dominik Amereller, Gero Kramer, Bernhard Grubmüller, Shahrokh Shariat, Alexander Haug, Marcus Hacker, Lukas Kenner, Gerda Egger

**Affiliations:** 1 Division of Nuclear Medicine, Department of Biomedical Imaging and Image-Guided Therapy, Medical University of Vienna, Vienna, Austria; 2 Christian Doppler Laboratory for Applied Metabolomics (CDLAM), Medical University of Vienna, Vienna, Austria; 3 Department of Pathology, Medical University of Vienna, Vienna, Austria; 4 Department of Urology, Medical University of Vienna, Vienna, Austria; 5 Department of Urology and Andrology, University Hospital Krems, Krems, Austria; 6 Karl Landsteiner University of Health Sciences, Krems, Austria; 7 Karl Landsteiner Institute of Urology and Andrology, Vienna, Austria; 8 Department of Urology, University of Texas Southwestern Medical Center, Dallas, TX USA; 9 Division of Urology, Department of Special Surgery, The University of Jordan, Amman, Jordan; 10 Department of Urology, Second Faculty of Medicine, Charles University, Prague, Czech Republic; 11 Department of Urology, Weill Cornell Medical College, New York, NY USA; 12 Clinical Institute of Pathology, Department for Experimental and Laboratory Animal Pathology, Medical University of Vienna, Vienna, Austria; 13 Comprehensive Cancer Center, Medical University Vienna, Vienna, Austria; 14 Unit of Laboratory Animal Pathology, University of Veterinary Medicine Vienna, Vienna, Austria; 15 Department of Molecular Biology, Umeå University, Umeå, Sweden

**Keywords:** cfDNA, DNA methylation, Epigenetics, Prostate cancer, PSMA, PET/CT

## Abstract

**Background:**

To validate the clinical utility of a previously identified circulating tumor DNA methylation marker (meth-ctDNA) panel for disease detection and survival outcomes, meth-ctDNA markers were compared to PSA levels and PSMA PET/CT findings in men with different stages of prostate cancer (PCa).

**Methods:**

122 PCa patients who underwent [⁶⁸Ga]Ga-PSMA-11 PET/CT and plasma sampling (03/2019–08/2021) were analyzed. cfDNA was extracted, and a panel of 8 individual meth-ctDNA markers was queried. PET scans were qualitatively and quantitatively assessed. PSA and meth-ctDNA markers were compared to PET findings, and their relative prognostic value was evaluated.

**Results:**

PSA discriminated best between negative and tumor-indicative PET scans in all (AUC 0.77) and hormone-sensitive (hsPC) patients (0.737). In castration-resistant PCa (CRPC), the meth-ctDNA marker *KLF8* performed best (AUC 0.824). *CHST11* differentiated best between non- and metastatic scans (AUC 0.705) overall, *KLF8* best in hsPC and CRPC (AUC 0.662, 0.85). Several meth-ctDNA markers correlated low to moderate with the tumor volume in all (5/8) and CRPC patients (6/8), while PSA levels correlated moderately to strongly with the tumor volume in all groups (all *p* < 0.001). CRPC overall survival was independently associated with *LDAH* and PSA (*p* = 0.0168, *p* < 0.001).

**Conclusion:**

The studied meth-ctDNA markers are promising for the minimally-invasive detection and prognostication of CRPC but do not allow for clinical characterization of hsPC. Prospective studies are warranted for their use in therapy response and outcome prediction in CRPC and potential incremental value for PCa monitoring in PSA-low settings.

**Supplementary Information:**

The online version contains supplementary material available at 10.1186/s13148-025-01811-5.

## Introduction

Prostate cancer (PCa) ranks as the second most frequent malignancy in men, causing approximately 400 000 annual deaths worldwide [1].

Due to a high degree of tumor heterogeneity, PCa survival rates vary from near-perfect 5-year overall survival (OS) rates in localized, hormone-sensitive (hsPC) to months in metastatic, castration-resistant PCa (CRPC) [2]. Disease outcomes are influenced by the clinical stage, determining the feasibility of curative approaches [3], and by the tumor's underlying molecular profiles [4], which continuously evolve in response to systemic interventions.

As these factors continually change throughout the disease progression [5], minimally-invasive means for reassessing tumor presence, advancement, and patient prognosis, are vital for adaptive and prompt clinical management [3] and rational trial design [6].

Well-known sensitivity and specificity limitations [7, 8] of prostate-specific antigen (PSA) testing for PCa detection, monitoring and prognosis [3], have prompted research into liquid biopsy assays as a source of PCa-specific biomarkers.

While most efforts have focused on genomic or transcriptomic analysis of circulating tumor DNA (ctDNA) or cells [9], epigenetic tumor DNA methylation modifications occur early, are stable and frequent [10], making them an attractive alternative source for high-sensitivity, PCa-specific, diagnostic and prognostic biomarkers [11–15].

We have previously also investigated the suitability of such ctDNA methylation markers for PCa-specific diagnostics and identified a panel of eight novel, high-potential ctDNA targets in the context of PCa [16]. As novel biomarkers are developed, their clinical characterization through contextualization with established diagnostic and prognostic approaches is key to informing their best use cases. We, therefore, sought to comparatively benchmark the previously identified ctDNA methylation marker panel against PSA levels using prostate-specific membrane antigen (PSMA) positron-emission-tomography/computed-tomography (PET/CT), the current imaging gold standard for PCa detection [17] and prognostic in itself [18, 19].

Our aim was to evaluate their relative predictive value for clinically actionable disease on PSMA imaging and their prognostic potential for OS outcomes.

We hypothesized that the meth-ctDNA markers are predictive of metastasis and survival outcomes in CRPC.

## Methods

### Study design

**Fig. 1:**
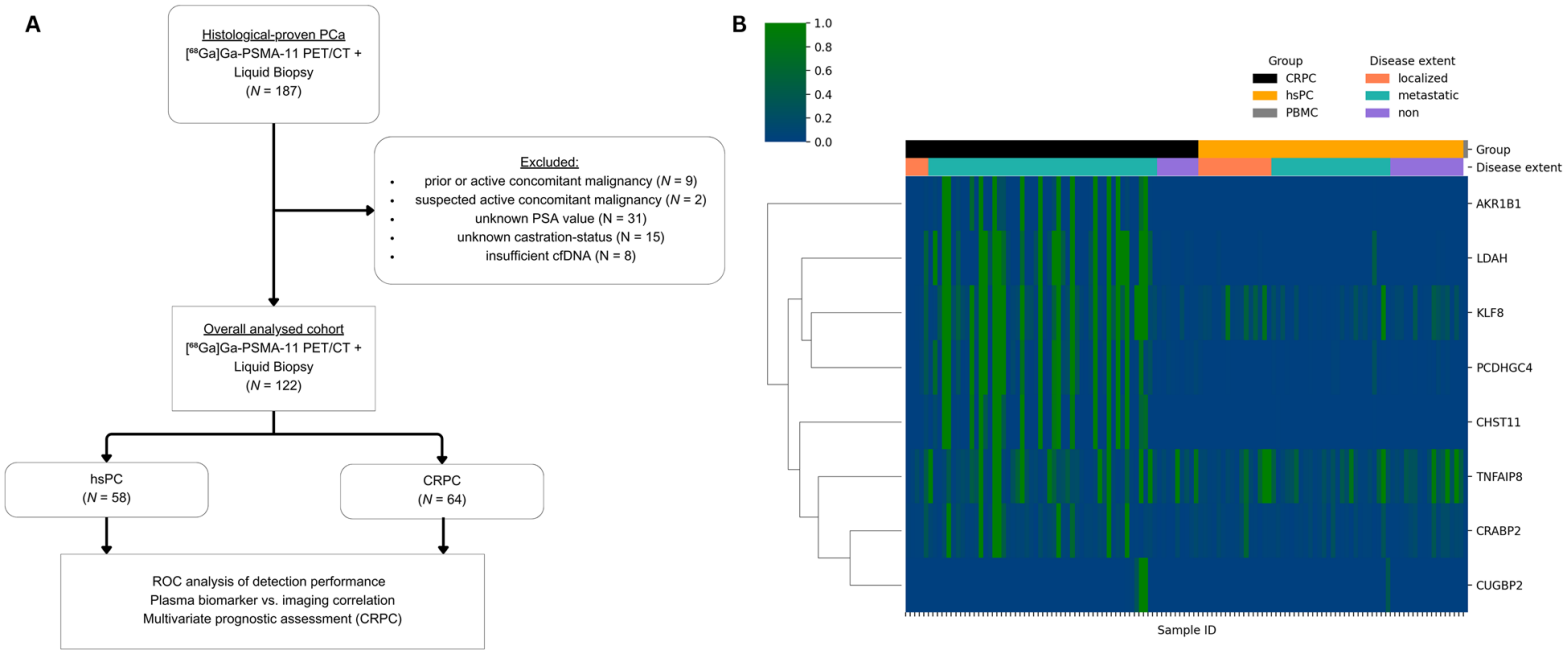
Consort diagram and unsupervised clustering analysis. **A** Illustrates the study's inclusion and exclusion criteria. **B** Imaging determined disease extent clustering of PMR-values according to castration status

From March 2019 to August 2021, a total of 187 men with histologically confirmed PCa underwent [⁶⁸Ga]Ga-PSMA-11 PET/CT imaging and provided blood samples [20] of which 122 patients were included in this analysis (Fig. 1). Recruitment followed an all-comers approach, with each participant providing written informed consent (IRB-ID: 1649/2016).

Retrospective data collection of PSA levels, castration state, treatment history and survival data (via the national health statistical service, censorization 27.02.2024) was conducted.

The endpoints were (a) differences and associations of meth-ctDNA markers and PSA levels with PSMA PET findings per castration status groups, (b) the prognostic association of meth-ctDNA markers levels, PSMA-TV and PSA with OS in CRPC patients. (Supplemental Material—Methods).

### Plasma sample collection, cfDNA extraction, quantification and methylation-sensitive restriction enzyme qPCR (MSRE-qPCR)

Sample collection and storage were previously described [20]. In brief, cell-free DNA (cfDNA) BCT tubes (Streck Inc., USA) were used for blood sampling prior to tracer injection. cfDNA was extracted from 4 ml of plasma employing QIAamp Circulating Nucleic Acid Kits (QIAGEN, the Netherlands) and quantified using a Qubit 3 Fluorometer (Invitrogen, USA).

MSRE-qPCR primers (Table S1) and workflow were previously described [16, 21]. Briefly, each cfDNA sample was split for methylation-sensitive enzymatic and mock digestion (75%:25%, respectively). Digestion reactions contained a mixture of four different MSREs, while mock digestions contained DNAse-free H_2_O instead of enzymes and served as DNA input normalization. Fully Methylated Human DNA (Zymo Research, USA) was used as methylation level reference control. All reactions were incubated at 37 °C for digestion, followed by a thermal inactivation, volume-reduction and multiplexed preamplification. qPCRs were performed using the Luna Universal qPCR Master Mix (New England Biolabs, USA) on a Biorad CFX96 Touch Real-Time PCR Detection System (Biorad Laboratories, USA).

Sample quantities were calculated using standard curves of genomic DNA from Cq values. Percentage of Methylation Ratios (PMR) was calculated using fully methylated DNA as the reference control. (Supplemental Material—Methods).

### Imaging protocol and image analysis

The imaging protocol and analysis was previously described [20]. In brief, imaging was performed on a Biograph TruePoint PET/CT scanner (Siemens Healthineers, Germany) an hour after i.v. [⁶⁸Ga]Ga-PSMA-11 injection (First acquisition: CT, Second acquisition PET (3–4 bed positions)). Next, images were reconstructed, scatter and attenuation corrected. Two nuclear medicine physicians used the Hybrid 3D software (v.4.0.0, Hermes Medical Solutions, Sweden) to qualitatively identify and semi-automatically delineate PSMA-expressing lesions by anatomical location to calculate the aggregated PSMA-positive tumor volume (PSMA-TV). PSMA-positive lesions were identified by qualitative PET scan analysis informed by the liver uptake, with lesions equal to or above liver uptake assumed malignant. Metastatic and non-metastatic disease state definitions were PSMA PET-based [22]*.* (Supplemental Material—Methods).

### Data analysis

Numeric variables are expressed as mean (± SD), discrete outcomes as absolute and relative (%) frequencies. Shapiro–Wilk and Levene’s tests assessed the normality and heteroskedasticity of continuous variables.

Data distribution informed, numeric variables were compared with the Mann–Whitney-U or Kruskal–Wallis tests, discrete outcomes with Chi-squared or Fisher’s exact tests.

Area under the receiver-operating-characteristic (AUC) curves (ROC) with 95% confidence intervals (CI) assessed the ability to predict PSMA PET findings. Correlations were evaluated using Spearman's coefficient. Dunn–Bonferronis corrections were used to adjust for multiple testing.

For the survival analysis, PMR, PSA and PSMA-TV values were dichotomized using the medians of the overall and the CPRC cohorts. Differences in OS between the respective high and low groups were compared using the non-parametric Logrank test, with censoring applied at the time of the last follow-up. Only univariate significant features were included in the multivariate Cox regression after testing data for multicollinearity and proportional hazards using the Belsley–Kuh–Welsch technique and Schoenfeld residuals. The alpha risk was set to 5% for all analyses. Statistical analysis was performed with EasyMedStat (version 3.32; www.easymedstat.com). (Supplemental Material—Methods).

## Results

### Cohort

122 PCa patients (age 70.9 ± 7.6 years; hsPC (N = 58), CRPC (N = 64)) who underwent plasma sample collection and [⁶⁸Ga]Ga-PSMA-11 PET/CT imaging were included in this study (Fig. 1). Patient characteristics are presented in Table 1.

**Table 1:** Demographic and clinical patient data

Variable	Total N = 122	hsPC N = 58	CRPC N = 64	*p*-Value
**Age [y]**	70.9 (± 7.6) Range: (49.0; 85.0)	70.0 (± 7.8) Range: (50.0; 85.0)	71.8 (± 7.3) Range: (49.0; 85.0)	0.201
**Tracer dose [Mbq]**	185.1 ± 19.5 Range: (134.0; 300.0)	186.1 (± 22.0) Range: (134.0; 300.0)	184.1 (± 17.0) Range: (149.0; 263.0)	0.802
**PSA [ng/mL]**	102.65 ± 448.03 Range: (0.01; 3689.0)	6.63 (± 12.29) Range: (0.18; 51.2)	189.67 (± 607.63) Range: (0.01; 3689.0)	< 0.001
**DNA methylation markers**				
*AKR1B1* [PMR]	2.82 (± 12.52) Range: (1e-06; 80.66)	0.00039 (± 0.00198) Range: (1e-06; 0.015)	5.38 (± 16.94) Range: (1e-06; 80.66)	< 0.001
*CHST11 *[PMR]	1.75 (± 8.6) Range: (0.0; 83.77)	0.00035 (± 0.0018) Range: (0.0; 0.0113)	3.34 (± 11.69) Range: (0.0; 83.77)	< 0.001
*CRABP2* [PMR]	0.49 (± 2.36) Range: (1.7e-05; 20.96)	0.0813 (± 0.102) Range: (0.000117; 0.597)	0.865 (± 3.22) Range: (1.7e-05; 20.96)	0.062
*CUGBP2* [PMR]*	0.13 (± 1.05) Range: (0.0; 10.75)	0.00637 (± 0.0475) Range: (0.0; 0.361)	0.247 (± 1.46) Range: (0.0; 10.75)	0.086
*KLF8 *[PMR]	3.3 (± 9.73) Range: (0.00317; 45.07)	0.238 (± 0.827) Range: (0.00317; 6.28)	6.08 (± 12.83) Range: (0.00664; 45.07)	0.001
*LDAH* [PMR]	3.41 (± 11.79) Range: (4.1e-05; 71.99)	0.0104 (± 0.0596) Range: (4.1e-05; 0.452)	6.5 (± 15.71) Range: (4.4e-05; 71.99)	< 0.001
*PCDHGC4 *[PMR]	2.23 (± 7.88) Range: (1e-06; 42.61)	0.0118 (± 0.0355) Range: (1e-06; 0.254)	4.25 (± 10.52) Range: (1e-06; 42.61)	< 0.001
*TNFAIP8 *[PMR]	1.06 (± 5.66) Range: (0.0; 58.36)	0.397 (± 0.675) Range: (5.5e-05; 3.69)	1.66 (± 7.77) Range: (0.0; 58.36)	0.737
**PSMA-TV [cm3]**	116.5 ± 305.5 Range: (0.0; 1597.7)	16.2 (± 86.5) Range: (0.0; 659.1)	207.4 (± 392.8) Range: (0.0; 1597.7)	< 0.001
**Disease extent**				< 0.001
Non	25 (20.49%)	16 (27.59%)	9 (14.06%)	
Localized	21 (17.21%)	16 (27.59%)	5 (7.81%)	
Metastatic	76 (62.3%)	26 (44.83%)	50 (78.12%)	
**PSMA-TV [cm3] per disease extent**				
Localized	4.5 (± 4.4) Range: (0.2; 15.4)	4.26 (± 4.1) Range: (0.2; 14.83)	5.28 (± 5.8) Range: (0.8; 15.38)	0.836
Metastatic	185.7 (± 370.5) Range: (0.2; 1597.7)	33.5 (± 128.4) Range: (0.2; 659.1)	264.9 (± 427.7) Range: (0.3; 1597.7)	< 0.001
**PSMA-positive lesions**				
Any lesion	97 (79.51%)	42 (72.41%)	55 (85.94%)	0.104
Prostate	39 (31.97%)	23 (39.66%)	16 (25.0%)	0.124
Lymph node	51 (41.8%)	19 (32.76%)	32 (50.0%)	0.081
Bone	51 (41.8%)	10 (17.24%)	41 (64.06%)	< 0.001
Organ	18 (14.75%)	4 (6.9%)	14 (21.88%)	0.023
**Hormonal therapies while PET**				< 0.001
Yes	48 (39.34%)	4 (6.9%)	44 (68.75%)	
No	68 (55.7%)	51 (87.93%)	17 (26.56%)	
**Cytotoxic therapies while PET**				0.403
Yes	4 (3.3%)	1 (1.72%)	3 (4.69%)	
No	114 (93.4%)	54 (93.1%)	60 (93.75%)	
**Therapies after PET**				< 0.001
Local	29 (39.19%)	22 (57.89%)	7 (19.44%)	
Local + ADT	5 (6.76%)	5 (13.16%)	0 (0.0%)	
ADT	17 (22.97%)	8 (21.05%)	9 (25.0%)	
CHT	3 (4.05%)	0 (0.0%)	3 (8.33%)	
CHT + ADT	2 (2.7%)	1 (2.63%)	1 (2.78%)	
Lu177	17 (22.97%)	1 (2.63%)	16 (44.44%)	
Study	1 (1.35%)	1 (2.63%)	0 (0.0%)	
**Time since diagnosis [y]**	5.44 ± 5.85 Range: (0.0; 21.0) N = 111	3.58 (± 4.67) Range: (0.0; 20.0) N = 55	7.27 (± 6.39) Range: (0.0; 21.0) N = 56	< 0.001
**Mean Follow-Up [m]**	24.88 ± 16.56	21.0 ± 16.33	29.15 ± 15.88	0.011
	Range: (0.0; 58.75)	Range: (0.43; 58.75)	Range: (0.0; 51.87)	

Qualitative data represented as numbers and percentages; Continuous data represented as mean, standard deviation and range; Group comparisons with respect to hsPC and CRPC. *Two CUGBP2 outlier values were excluded from the analysis

### ctDNA methylation marker and PSA level’s discriminatory value to distinguish between positive and negative PSMA PET

**Fig. 2:**
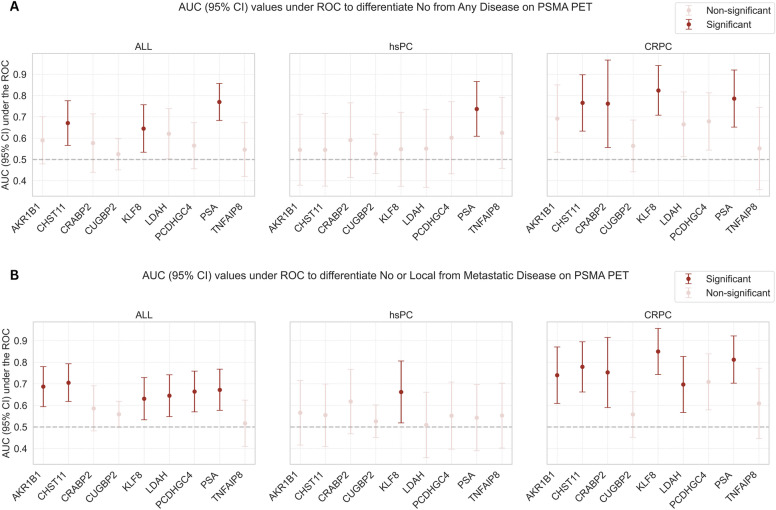
AUC (95% CI) values under the ROC depict the discriminatory value of the ctDNA PMR and PSA values to distinguish between **A** No and any tumor-indicative lesion and **B** No or Local and metastasis-indicative lesions on PSMA PET. Significant differing variables between globally positive and negative as well as non- and metastatic PSMA scans in dark red

In the overall cohort, significant differences between patients with negative and tumor-indicative PSMA scans were found for 2/8 (25%) of the analyzed meth-ctDNA markers (*CHST11* (*p* = 0.007), *KLF8* (*p* = 0.026)) and PSA (*p* < 0.001), with the highest AUC for PSA (AUC 0.77, CI = [0.683; 0.857]) (Figs. 2A, S2, Tables S2, S4, S17). In the hsPC cohort, no meth-ctDNA marker and merely PSA differed significantly (*p* = 0.006) between patients with positive and negative PSMA scans, which exhibited an AUC of 0.737 (CI = [0.609; 0.866]) (Fig. 2A, S3, Tables S2, S5, S17). While in CRPC patients 3/8 (32.5%) of the meth-ctDNA markers (*CHST11* (*p* = 0.011), *CRABP2* (*p* = 0.013), *KLF8* (*p* = 0.002)) and PSA (*p* = 0.006) differed significantly with *KLF8* exhibiting the highest AUC of 0.824 (CI = [0.652; 0.92]) (Figs. 2A, S4, Tables S2, S6, S17).

### ctDNA methylation marker and PSA level’s discriminatory value to identify metastatic disease on PSMA PET

In all patients, 5/8 (62.5%) of the analyzed meth-ctDNA markers (*AKR1B1* (*p* < 0.001), *CHST11* (*p* < 0.001), *KLF8* (*p* = 0.016), *LDAH* (*p* = 0.007), *PCDHGC4* (*p* = 0.002)) and PSA levels (*p* < 0.001) differed significantly between patients with non-metastatic and metastatic disease on the PSMA scans, with the highest AUC under the ROC achieved by *CHST11* (AUC—0.705, CI = [0.618; 0.793]) (Figs. 2B, S5, Tables S2, S7–9, S18).

In hsPC patients, only 1/8 (12.5%) of the meth-ctDNA markers, specifically *KLF8* (*p* = 0.035), differed significantly between patients with non-metastatic and metastatic PSMA scans, while PSA levels did not, with an AUC of *KLF8* (AUC—0.662, CI = [0.519; 0.806]) (Figs. 2B, S7, Tables S2, S10–12, S18).

In the CRPC group, 6/8 (75%) of meth-ctDNA markers (*AKR1B1* (*p* = 0.007), *CHST11* (*p* = 0.001), *CRABP2* (*p* = 0.004), *KLF8* (*p* < 0.001), *LDAH* (*p* < 0.026), *PCDHGC4* (*p* = 0.018)) and PSA levels (*p* < 0.001) differed significantly between non-metastatic and metastatic PSMA scans, with the highest AUC achieved by *KLF8* (AUC 0.85, CI = [0.743; 0.957]) (Figs. 2B, S9, Tables S2, S13–15, S18).

### Relationship of ctDNA methylation marker and PSA levels with the PET PSMA-TV

Positive associations between meth-ctDNA markers and PSMA-TV were found in the overall cohort and CRPC group; however, not in the hsPC cohort. In the overall cohort, low positive correlations were observed for *AKR1B1* (r = 0.4, *p* < 0.001), *CHST11* (r = 0.44, *p* < 0.001), *KLF8* (r = 0.37, *p* < 0.001), *LDAH* (r = 0.44, *p* < 0.001) and *PCDHGC4* (r = 0.46, *p* < 0.001) and PSMA-TV. In the CRPC group, moderate positive correlations were observed for *AKR1B1* (r = 0.56, p < 0.001), *CHST11* (r = 0.58, *p* < 0.001), *CRABP2* (r = 0.44, *p* = 0.002), *KLF8* (r = 0.62, *p* < 0.001), *LDAH* (r = 0.57, *p* < 0.001) and *PCDHGC4* (r = 0.51, *p* < 0.001) and PSMA-TV. No significant negative correlation was found between any meth-ctDNA marker and PSMA-TV in any group.

**Fig. 3:**
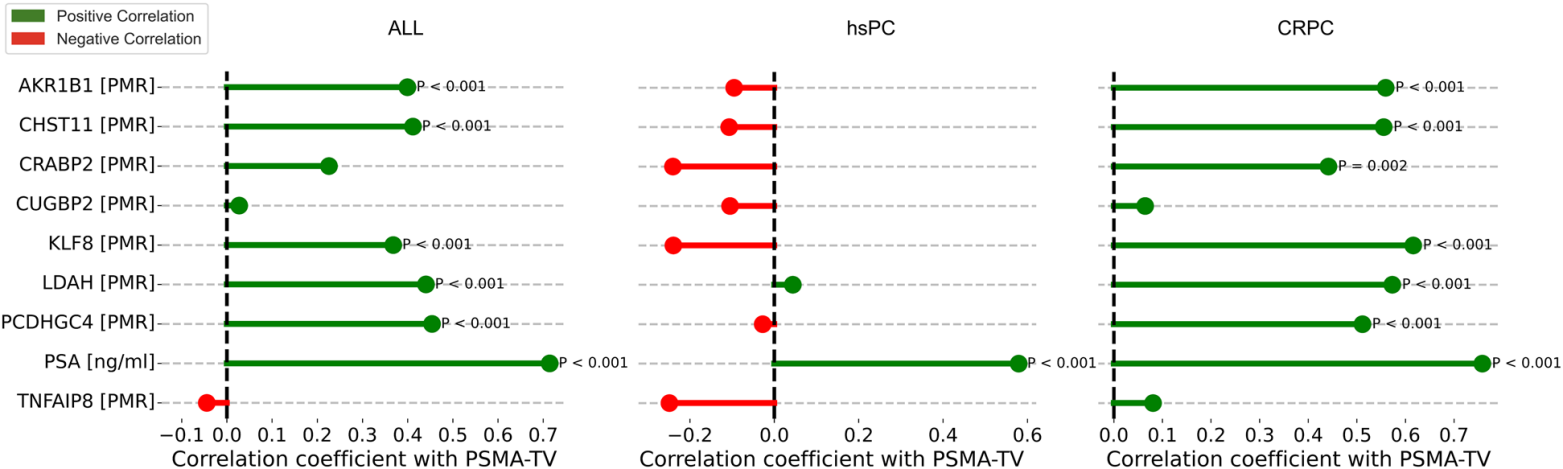
Relationship of meth-ctDNA PMR and PSA values with the PSMA-TV according to castration statuses. Red bars indicate negative correlations, green bars positive correlations. Only significant p-values after Bonferroni multiple-testing adjustments are displayed

Moderate to strong positive correlations were observed between PSA levels with PSMA-TV in all groups (r = 0.71 (overall), r = 0.58 (hsPC), r = 0.76 (CRPC), all *p* < 0.001). A visual overview of the results is shown in Fig. 3 and listed in Table S18.

### Survival analysis

Of the 64 patients included in the OS analysis, 28 patients (43.75%) events were observed, while 36 patients (56.25%) were censored at the last follow-up. The median duration of follow-up was 19.92 months (IQR 27.62). At 12 months, the OS was 71.9% (CI = [59.1, 81.3]) and at 24 months, the OS was 62.5% (CI = [49.5, 73.1]).

**Fig. 4:**
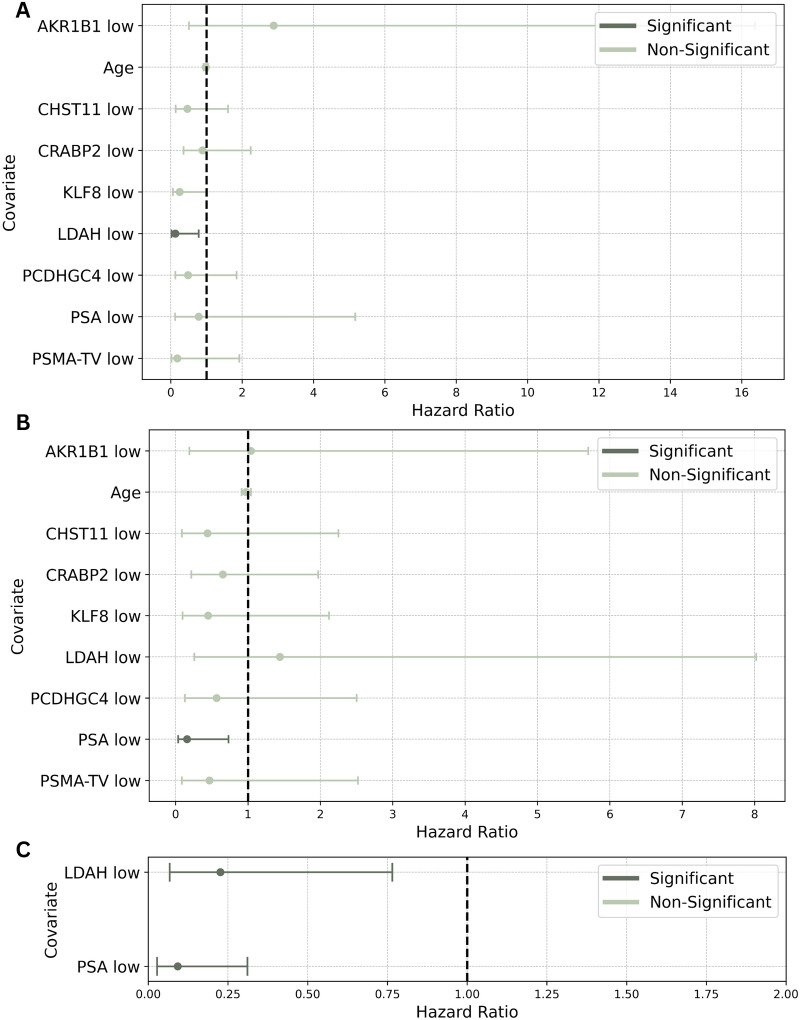
Forest plots depicting the HR of the explanatory DNA methylation markers, PSA and PSMA-TV values and age. High and low group stratification by median of the overall cohort **A** and of the CRPC group **B**. **C** depicts the HRs of significant covariates from analyses **A** and **B**, suggesting an independent association of *LDAH* and PSA levels with OS in CRPC patients

Patients with high and low *LDAH* values (stratified on the overall cohort) (hazard ratio (HR) = 7.91, CI = [1.28, 49.05], *p* = 0.0263) (Fig. 4A, Table 2) and patients with high and low PSA levels (stratified on the CRPC cohort) (HR = 6.29, CI = [1.37, 28.86], *p* = 0.0181]) (Fig. 4B, Table 2) had significant hazard differences. Both *LDAH* and PSA levels remained independently associated with OS in CRPC patients when analyzed in a comparative multivariate analysis (HR = 4.42, CI = [1.31, 14.94], *p* = 0.0168 and HR = 10.82, CI = [3.21, 36.45], *p* < 0.001, respectively) (Fig. 4C, Table 2). For the univariate log-rank analysis results, please refer to Figs S11, S12 and Tables S19, 20.

**Table 2:** Multivariate Cox regression of the binarily stratified explanatory DNA methylation markers, PSA and PSMA-TV values and age

Covariate	Hazard Ratio [95% CI] [overall]	*p*-Value	Hazard Ratio [95% CI] [CRPC]	*p*-Value	Hazard Ratio [95% CI] [comparative]	*p*-Value
**Age**						
Risk for each 1-unit increase	0.978 [0.917; 1.04]	0.499	0.973 [0.912; 1.04]	0.398	−	−
***AKR1B1***						
high	0.347 [0.0611; 1.98]	0.233	0.96 [0.175; 5.25]	0.962	−	−
low	2.88 [0.506; 16.37]	0.233	1.04 [0.19; 5.7]	0.962	−	−
***CHST11***						
high	2.16 [0.625; 7.45]	0.224	2.28 [0.444; 11.68]	0.324	−	−
low	0.463 [0.134; 1.6]	0.224	0.439 [0.0856; 2.25]	0.324	−	−
***CRABP2***						
high	1.12 [0.446; 2.82]	0.807	1.53 [0.507; 4.63]	0.449	−	−
low	0.891 [0.355; 2.24]	0.807	0.653 [0.216; 1.97]	0.449	−	−
***KLF8***						
high	4.06 [0.984; 16.72]	0.0527	2.22 [0.472; 10.48]	0.313	−	−
low	0.247 [0.0598; 1.02]	0.0527	0.45 [0.0954; 2.12]	0.313	−	−
***LDAH***						
high	7.91 [1.28; 49.05]	0.0263	0.694 [0.125; 3.86]	0.676	4.42 [1.31; 14.94]	0.0168
low	0.126 [0.0204; 0.783]	0.0263	1.44 [0.259; 8.02]	0.676	0.226 [0.067; 0.765]	0.0168
***PCDHGC4***						
high	2.08 [0.545; 7.92]	0.285	1.77 [0.401; 7.78]	0.452	−	−
low	0.482 [0.126; 1.84]	0.285	0.566 [0.128; 2.5]	0.452	−	−
**PSA**						
high	1.28 [0.193; 8.44]	0.799	6.29 [1.37; 28.86]	0.0181	10.82 [3.21; 36.45]	0.000012
low	0.783 [0.119; 5.17]	0.799	0.159 [0.0346; 0.731]	0.0181	0.092 [0.027; 0.311]	0.000012
**PSMA-TV**						
high	5.46 [0.52; 57.27]	0.157	2.14 [0.396; 11.51]	0.377	−	−
low	0.183 [0.0175; 1.92]	0.157	0.468 [0.0869; 2.52]	0.377	−	−

Group stratification by median of the overall cohort ([overall]), and CRPC group ([CRPC]), except for age in years. Results for the comparative regression analysis incorporating significant findings of overall and CRPC group stratified covariates in the two rightmost columns

## Discussion

Liquid biopsy approaches hold the promise to advance PCa management through minimally-invasive, repeatable molecular cancer profiling to enable more accurate disease diagnosis, monitoring, and prognostication. Particularly, epigenetic ctDNA methylation modifications are an attractive source of PCa-specific biomarkers, as they occur early, are stable and more abundant than somatic gene alterations [9–11]. We previously investigated [16] the suitability of such ctDNA methylation markers for PCa-specific diagnosis and prognosis and identified a panel of eight high-potential targets. To validate their clinical utility, we compared these markers against PSA levels and PSMA PET/CT findings to assess their relative predictive value for clinically actionable disease presence and survival outcomes.

In the setting of local or oligometastatic hsPC, disease-, risk- and extent-specific biomarkers could enable accurate primary screening practices [7], refine active surveillance strategies, and help inform clinical management in curative and salvage settings [23].

In our hsPC group, however, no meth-ctDNA marker accurately discriminated between patients with no or any disease on imaging, unlike PSA, while only *KLF8* differed between hsPC patients with non- and metastatic PSMA scans (Fig. 2). Upon closer examination of the *KLF8* value distribution between the non- and metastatic groups (Figure S7), however, no definite differentiation for the majority of patients was apparent, suggesting that the observed difference would not broadly translate into future clinical applicability. Similarly, no meth-ctDNA marker exhibited any tangible relationship with PSMA-TV in patients with hsPC, while PSA correlated with the overall tumor load (Fig. 3).

This is in line with our previous observations [16] and various reports [9, 24] of low ctDNA abundance using genomic [25] or epigenetic [12–14] modes of analysis in localized and low-volume metastatic hsPC disease. Bjerre et al. [12] investigated the diagnostic and prognostic potential of a three-gene methylation ctDNA signature consisting of *DOCK2, HAPLN3,* and *FBXO30*. This signature was able to differentiate de novo, primarily high-volume metastatic PCa; however, was not able to differentiate between healthy controls, benign prostatic hyperplasia and localized PCa. Similarly, large-scale efforts by the company GRAIL Inc., which developed a ctDNA methylation-based machine learning enabled multi-cancer screening test on more than 6000 plasma samples using 100 000 methylated DNA regions, did not yield favorable PCa screening test statistics in a validation cohort reported on by Klein et al. [14]. The authors concluded that this might be due to the inclusion of too many localized PCas, which corroborates the hypothesis that too little ctDNA is shed in early disease stages and therefore alternative analytic approaches, such as proteomics [26], might yield more promising biomarkers in this setting in the future. However, a bias toward the investigated meth-ctDNA markers being more specific for CRPC disease cannot be fully excluded.

Advanced CRPC are heterogeneous cancers [27] characterized by variable responses to systemic therapies and outcomes. Minimizing therapeutic downtime by identifying progression early is key to maximizing outcomes. However, evidence suggests a frequent disconnection between PSA dynamics and radiographic responses [8] or survival outcomes [28–30], and reliable OS-surrogate intermediate clinical endpoints (ICEs) in mCRPC are missing [6].

In our CRPC group, both PSA and several meth-ctDNA markers demonstrated similarly strong discriminatory abilities to differentiate between patients with no or any lesion on imaging and non- and metastatic disease presence (Fig. 2). Particularly, *KLF8*, a transcription factor linked to cancer invasion and metastasis [31], demonstrated strong discriminatory potential and followed response dynamics in patients undergoing various systemic therapies [16]. Analogously, both meth-ctDNA marker PMR and PSA levels were robustly associated with the underlying tumor load in the CRPC group (Fig. 3), with PSA exhibiting a slightly stronger correlation with PSMA-TV. Similarly, Büttner et al. [15] investigated the potential of the two ctDNA methylation markers *SHOX2* and *SEPT9* using a methylation-specific PCR approach in a pilot cohort of advanced PCa. They found that both methylation markers correlated with imaging-based tumor burden dynamics under therapy, however, PSA did not consistently follow suit. This corroborates our previous finding that meth-ctDNA markers show potential for detecting and monitoring disease dynamics [16], potentially mitigating known challenges of outcome-discordant PSA-based monitoring [28–30]. However, as PSA levels were generally at least on par with the meth-ctDNA levels in our CRPC cohort, a possible incremental value of the studied meth-ctDNA markers should be investigated in prospectively collected cohorts with known advanced, low-PSA CRPC.

As CRPC is associated with high mortality [2] and OS surrogacy ICEs are needed [6], we sought to investigate the comparative predictive value of the investigated meth-ctDNA markers, PSMA-TV and PSA for OS outcomes. Therefore, following an evidence-based variable selection approach, we conducted a multivariate Cox regression analysis (Fig. 4), using the median variable values of the overall and CRPC as stratifying cutoffs. *LDAH* (overall cutoff), and expectedly [32], PSA levels (CRPC cutoff) were significantly associated with OS and remained independently associated with OS when tested in a comparative fashion, indicating that *LDAH* could be a novel potential ICE for OS surrogacy [6] and should be investigated in future trials in the CRPC setting.

Several limitations of our study merit discussion.

First, as a translational clinical study, no novel mechanistic insights into the biological role of the meth-ctDNA markers was generated, which limits mechanistic interpretation. However, several meth-ctDNA markers have been implicated in different hallmark processes of cancer, supporting their biological validity bibliographically. For instance, *AKR1B1*, frequently overexpressed in several cancers, plays diverse roles in cell cycle regulation and epithelial-mesenchymal transition (EMT). Its promoter methylation has also been suggested as a diagnostic marker in breast cancer, and its inhibition has been shown to exhibit anti-neoplastic effects [33]. Similarly, *KLF8* has inter alia been implicated in EMT and invasion [34] and DNA repair [35] in breast cancer. The loss of *LDAH*, a gene coding for a lipid hydrolase, has been linked to an increased risk of PCa in vivo and in vitro [36], and altered methylation patterns of clustered protocadherins, including *PCDHGC4*, have been observed in various solid cancers [37], while *CHST11* [38, 39] and *CRABP2* [40] have been suggested as potential diagnostic and therapeutic targets due to their involvement in several processes ranging from cancer cell stemness, EMT, cell proliferation, cell cycle and drug resistance in different entities.

Second, the research is limited due to several factors, namely, its small cohort size, monocentricity and retrospective nature, which can all negatively influence generalizability.

The small cohort size could lead to confounding influences and insufficient statistical power, negatively influencing generalizability, particularly in the subgroup analyses.

The mono-centric design did not allow for independent validation of the PSMA-TV correlations with meth-ctDNA, which we tried to mitigate using a multiple-testing correction to avoid false discoveries.

Its retrospective nature, makes us prone to recall and potential selection bias, which we mitigated by excluding patients with inconclusive records and employing an all-comer recruitment strategy. Next, while PSMA PET/CT offers the highest detection for metastatic disease [17], false negative lesions can occur, as signified by one patient in our cohort with a PSMA-negative, biopsy-proven pulmonary PCa metastasis. Further, due to the exploratory study design, which aimed at gaining a contextualizing perspective across the whole spectrum of disease, the utilized cohort was biological and therapeutic heterogeneous partially limiting the direct clinical interpretability.

Despite the study's limitations, its strengths also merit acknowledgment. Contemporaneous tracer injection and plasma sampling ensured biological synchronicity for an optimal comparative perspective, and the balanced inclusion of castration statuses allowed for an informative perspective across the disease spectrum.

As the investigated meth-ctDNA markers partially show great potential for accurate, minimally-invasive diagnosis of mCRPC and survival prognostication, their applicability for systemic therapy response prediction, as ICEs for outcome surrogacy and their incremental value for disease monitoring in PSA-low advanced PCa should be investigated in future prospective trials.

## Conclusion

This study identified ctDNA methylation markers that appear accurate for the minimally-invasive detection and outcome prognostication for advanced, castration-resistant disease but do not seem suitable for clinical characterization of hormone-sensitive PCa. This warrants further prospective studies for their potential applicability for systemic therapy response and outcome prediction in advanced CRPC and their incremental value for disease monitoring in PSA-low advanced PCa.

## Supplementary Information

Additional file1 (PDF 2506 KB)

## Data Availability

Upon request.
