# Supplementary material for: Imaging and outcome correlates of ctDNA methylation markers in prostate cancer: a comparative, cross-sectional [⁶⁸Ga]Ga-PSMA-11 PET/CT study
Source: Clin Epigenetics. 2025 Feb 25;17:36. doi: 10.1186/s13148-025-01811-5 (PMC11863674; doi:10.1186/s13148-025-01811-5)
Supplement: Supplementary file 1 — Additional file1 (PDF 2506 KB) [file 13148_2025_1811_MOESM1_ESM.pdf]

Supplemental Material & Data

Imaging and outcome correlates of PCa-specific ctDNA methylation markers in Prostate Cancer: A comparative, cross-sectional [^{68}Ga]Ga-PSMA-11 PET/CT study

Kilian Kluge[1,2*], Vincent Lotz[3], Holger Einspieler[1], David Haberl[1,2], Clemens Spielvogel[1], Dominik Amereller[1], Gero Kramer[4], Bernhard Grubmüller[5-6], Shahrokh Shariat[4,7-11], Alexander Haug[12], Marcus Hacker[1,14], Lukas Kenner[2,3,13-16], Gerda Egger[3,14]

1 Department of Biomedical Imaging and Image-Guided Therapy, Division of Nuclear Medicine, Medical University of Vienna, Vienna, Austria
2 Christian Doppler Laboratory for Applied Metabolomics (CDLAM), Medical University of Vienna, Vienna, Austria
3 Department of Pathology, Medical University of Vienna, Vienna, Austria
4 Department of Urology, Medical University of Vienna, Austria
5 Department of Urology and Andrology, University Hospital Krems, 3500 Krems, Austria
6 Karl Landsteiner University of Health Sciences, 3500 Krems, Austria
7 Karl Landsteiner Institute of Urology and Andrology, Vienna, Austria
8 Department of Urology, University of Texas Southwestern Medical Center, Dallas, Texas, USA
9 Division of Urology, Department of Special Surgery, The University of Jordan, Amman, Jordan
10 Department of Urology, Second Faculty of Medicine, Charles University, Prague, Czech Republic
11 Department of Urology, Weill Cornell Medical College, New York, New York, USA
12 Vienna, Austria
13 Clinical Institute of Pathology, Department for Experimental and Laboratory Animal Pathology, Medical University of Vienna, Vienna, Austria
14 Comprehensive Cancer Center, Medical University Vienna, Vienna, Austria
15 Unit of Laboratory Animal Pathology, University of Veterinary Medicine Vienna, Vienna, Austria
16 Department of Molecular Biology, Umeå University, Umeå, Sweden

*First Author
Kilian Kluge, Division of Nuclear Medicine, Medical University of Vienna, Währinger Gürtel 18-20, 1090 Vienna, Austria
kilian.kluge@meduniwien.ac.at
ORCID: 0000-0002-4224-7314

#Correspondence:
Gerda Egger, Department of Pathology, Medical University of Vienna, Währinger Gürtel 18-20, 1090 Vienna, Austria
gerda.egger@meduniwien.ac.at
ORCID: 0000-0003-2489-155X

Table of Contents

Table of Contents **2**
Methods **3**
Study design 3
Plasma sample collection, cfDNA extraction and quantification 3
Methylation-sensitive restriction enzyme qPCR (MSRE-qPCR) 4
Imaging protocol and image analysis 5
Statistical analysis 6
Results **7**
Overview of significant differences and correlations between ctDNA methylation markers or PSA levels and PSMA imaging findings 7
Meth-DNA PMR and PSA levels according to castration status 8
Meth-DNA PMR and PSA levels according to global PET positivity in all patients 9
Meth-DNA PMR and PSA levels according to global PET positivity in the hsPC patients 10
Meth-DNA PMR and PSA levels according to global PET positivity in the CRPC patients 11
Meth-DNA PMR and PSA levels according to PSMA PET disease extent in all patients 12
Meth-DNA PMR and PSA levels according to PSMA PET disease extent in hsPC patients 15
Meth-DNA PMR and PSA levels according to PSMA PET disease extent in CRPC patients 17
Table S17: AUC [95% CI] values of ROC analysis to distinguish between non-metastatic and metastatic disease on PSMA PET imaging. Abbreviations: hsPC - hormone-sensitive prostate cancer, CRPC - castration-resistant prostate cancer 20
Meth-DNA PMR and PSA levels correlation with PSMA-TV according to castration group 21
Univariate log-rank test comparison of survival distributions 22

Methods

Study design

Between March 2019 to August 2021, 187 men with histologically confirmed PCa underwent [^{68}Ga]Ga-PSMA-11 PET/CT imaging and blood sample collection. An all-comer recruitment strategy was used, approaching all men referred to PSMA PET imaging. This study was approved by the IRB of the Medical University of Vienna (ID: 1649/2016). All patients gave their written informed consent for PET imaging, sample collection and associated analysis. Subsequently, data collection such as prostate-specific antigen (PSA) levels and treatment regimens before, during and after imaging was retrospectively conducted. PCas not exposed to any antiandrogen or which did not advance in a castrate environment were classified as hsPC, while PCas which progressed in a castrate environment were deemed CRPC [1]. Survival data was sourced through the national health statistical service, Statistik Austria (censorization date 27.02.2024).

In this analysis, only patients with complete PSA level and castration status records were included, while patients with prior or concomitant malignancies other than PCa and samples with less than 3ng of cfDNA for analysis were excluded (Figure 1), resulting in 122 analysed patients. The endpoints were a) differences and associations of PCa-specific ctDNA methylation markers and PSA levels with PSMA-PET findings per castration status groups and b) prognostic association of PCa-specific ctDNA methylation marker levels, PSMA-TV and PSA with overall survival in CRPC patients.

Plasma sample collection, cfDNA extraction and quantification

Blood samples were collected in Cell-Free DNA BCT tubes (Streck Inc., Nebraska, USA, Ref. 230470) directly before tracer application, followed by cellular debris removal using a double spin protocol (first spin 2000g for 20 min., second spin 3200g for 30min.). Next, the plasma samples were stored at -80°C until further processing. The QIAamp Circulating

Nucleic Acid Kits (QIAGEN, Venlo, Netherlands, Ref. 55114) were used to extract cfDNA from 4 ml of plasma, adhering to the manufacturer's protocol. cfDNA was eluted in 40µL of DNAse-free water (dfH20). Afterwards, cfDNA was stored at -20°C till further analysis. cfDNA quantification was performed on a Qubit 3 Fluorometer (Invitrogen, California, USA) using the high-sensitivity dsDNA Quantification Assay (Invitrogen, Ref. Q33230), according to the manufacturer's instructions.

Methylation-sensitive restriction enzyme qPCR (MSRE-qPCR)

The utilized MSRE-qPCR primers and workflow were previously described by Dillinger [2] and Beikircher et al [3], respectively. Briefly, for each sample, 3-10ng cfDNA was allocated in a 75% to 25% split ratio to methylation-sensitive enzymatic digestion and mock digestion reactions. Digestion reactions contained a mixture á 2 Units of the following enzymes, AciI (New England Biolabs (NEB), Massachusetts, USA, Ref. R0551), HpaII (Thermo Fisher Scientific (TF), Massachusetts, USA, Ref. ER0511), HpyCH4IV (NEB, Ref. R0619) and Hin6I (TF, Ref. ER0481) in ultrapure water and 10x Tango Buffer (TF). Mock digested contained dfH20 instead of enzymes and served for DNA input normalisation. Fully Methylated Human DNA (fmDNA) (Zymo Research, California, USA, Ref. D5013) was used as methylation level reference control. Next, all reactions were incubated at 37°C for 16 hours for digestion, followed by thermal inactivation of enzymes at 65°C for 20 min on the Biorad C1000 Thermo Cycler system (Biorad Laboratories, California, USA). Subsequently, the processed samples were volume reduced using the Microcon-10kDa Centrifugal Filters (Merck KGaA, Darmstadt, Germany, Ref. MRCPRT010), followed by a multiplexed PCR preamplification using the 2x TaqMan Preamplification Master Mix (TF, Ref. 4391128) in a 50nM mixture of pooled primer pairs. Afterwards, qPCRs were performed as duplicates using the Luna Universal qPCR Master Mix (NEB, Ref. MLP965) in white 96-well plates (Biorad Laboratories, Ref. MLP9651), according to the manufacturer specifications on a Biorad CFX96 Touch Real-Time PCR Detection system (Biorad Laboratories).

From the yielded Cq values, sample quantities (SQ) were calculated using 4-fold, 3-step dilution series standard curves of genomic DNA (93.8ng/run—1.47 ng/run). Percentage of Methylation Ratios (PMR) were calculated for each sample using the following formula.

$$PMR = 100 * \left(\frac{SQ\ (gene)\ digested\ sample}{SQ\ (gene)\ mock\ digested\ sample)}\right) / \left(\frac{SQ\ (gene)\ fmDNA\ digested)}{SQ\ (gene)\ fmDNA\ undigested)}\right)$$

Target	Forward/Sense	Revers/Antisense
AKR1B1	GCTTCTTCCGCCTGGTCCTAGTGG	TTCGCTTTCCCACCAGATACAGCA
CHST11	GGGGTGCGAGGGAAAGTTTGG	GACCCGCGTTACCCGGAAGG
CRABP2	GGCCGGAAACCGCAGAGGAG	GACAGGCCGGGACGGTTCCT
CUGBP2	GCACCTGTCCCTGCCCGTCT	CGACCATCATGTCCGGGAGGA
KLF8	GCGGTGAAGGGATCCTCTTGTGG	CGGGGGTGGGGGCTATGAAA
LDAH	CTGGGAAGGCGGCACGAGAC	CAGCTCCGGCGCGGTACTGT
PCDHGC4	ACCCCGCCACCAGCAAAAAC	GCCCCAAACAGCCACAGCAG
TNFAIP8	CAGAGCGAACTTGCGGCTCGT	CGGCCCCCAGGAGAAACCAG

Table S1: Forward and Reverse primer sequences used in the MSRE-qPCR cfDNA analysis

Imaging protocol and image analysis

[^{68}Ga]Ga-PSMA-11 was intravenously injected (mean tracer dose: 185.1 MBq ± 19.5 SD), with subsequent patient scanning on Biograph TruePoint PET/CT system (Siemens Healthineers, Erlangen, Germany) one-hour p.i. from the skull base to the upper femur. First, CT scans were acquired with intravenous contrast, except contraindications existed at 120kv and 230 mAs. Next, PET scans were acquired in 3-4 bed positions (PET matrix size 168x168), followed by iterative reconstruction using a point-spread-function-based algorithm (Siemens Healthineers) and subsequently scatter and attenuation corrected based on the CT component.

PSMA PET scans were analysed by two nuclear medicine physicians on a dedicated workstation using the Hybrid 3D software (v4.0.0, Hermes Medical Solutions, Stockholm, Sweden), delineating all PSMA-expressing tumors and metastasis and labelling them according to their anatomical locations. PSMA-positive lesions were identified by qualitative PET scan analysis informed by the liver uptake, with lesions equal to or above liver uptake

assumed malignant. Metastatic and non-metastatic disease state definitions were PSMA PET-based [4].

Statistical analysis

Numeric variables are expressed as mean (±SD) and discrete outcomes as absolute and relative (%) frequencies. Shapiro-Wilk and Levene's tests were used to assess the normality and heteroskedasticity of continuous variables, respectively.

Numeric variables according to castration status, PET positivity and binary disease extent groups were compared with unpaired t-test, Welch t-test or Mann-Whitney U test according to the underlying data distribution. Discrete outcomes were compared with chi-squared or Fisher's exact test accordingly. All tests were two-tailed. Continuous variables according to the three classes of disease extent were compared using Kruskal-Wallis tests according to data distribution, followed by Dunn-Bonferroni's multiple-testing correction in case the null hypothesis was rejected. We used Area under the Receiver-Operating-Characteristic (AUC) curves (ROC) with 95% confidence intervals (CI) to assess the ability to predict PET positivity and binary disease extent.

Spearman's coefficient was used to assess the correlation between PMR vales or PSA levels and PSMA-TV according to the castration group, using the Dunn-Bonferronis test for multiple testing corrections. Correlations were judged very strong from 1 to 0.9, strong from 0.9 to 0.7, moderate from 0.7 to 0.5, low from 0.5 to 0.3 and poor from 0.3 to 0.

For the survival analysis, PMR, PSA and PSMA-TV vales were dichotomised using the medians of the overall and the CPRC cohorts. Next, the Logrank non-parametric test was used to compare survival differences between the respective median-stratified high and low groups. Following an evidence-based assessment strategy, only univariate significant features were included in the multivariate Cox regression alongside patient age as a potential confounder of the multivariate survival analysis. Data were tested for multicollinearity and proportional hazards using the Belsley-Kuh-Welsch technique and

Schoenfeld residuals, respectively. The alpha risk was set to 5% for all analyses. Statistical analysis was performed with EasyMedStat (version 3.32; www.easymedstat.com).

Results

Overview of significant differences and correlations between ctDNA methylation markers or PSA levels and PSMA imaging findings

Target	AKR1B1	CHST11	CRABP2	CUGBP2	KLF8	LDAH	PCDHGC4	TNFAIP8	PSA
**Group [hsPC vs CRPC]**	yes	yes	no	no	yes	yes	yes	no	yes
**Global PET pos [ALL]**	no	yes	no	no	yes	no	no	no	yes
**Global PET pos [hsPC]**	no	no	no	no	no	no	no	no	yes
**Global PET pos [CRPC]**	no	yes	yes	no	yes	no	no	no	yes
**Non/Local vs Metastatic PET [ALL]**	yes	yes	no	no	yes	yes	yes	no	yes
**Non/Local vs Metastatic PET [hsPC]**	no	no	no	no	yes	no	no	no	no
**Non/Local vs Metastatic PET [CRPC]**	yes	yes	yes	no	yes	yes	no	no	yes
**PSMA-TV [cm3] [ALL] - Correlation**	yes	yes	no	no	yes	yes	yes	no	yes
**PSMA-TV [cm3] [hsPC] - Correlation**	no	no	no	no	no	no	no	no	yes
**PSMA-TV [cm3] [CRPC] - Correlation**	yes	yes	yes	no	yes	yes	yes	no	yes

Table S2: Overview of significant differences in ctDNA methylation markers and PSA levels according to disease group (group), global PSMA positivity (Global PET pos), disease extent on PSMA PET (Non/Local vs Metastatic PET), as well as significant correlations between PSMA-TV and ctDNA methylation markers and PSA levels. ctDNA methylation marker levels as relative PMR values, PSA in ng/ml.

Meth-DNA PMR and PSA levels according to castration status

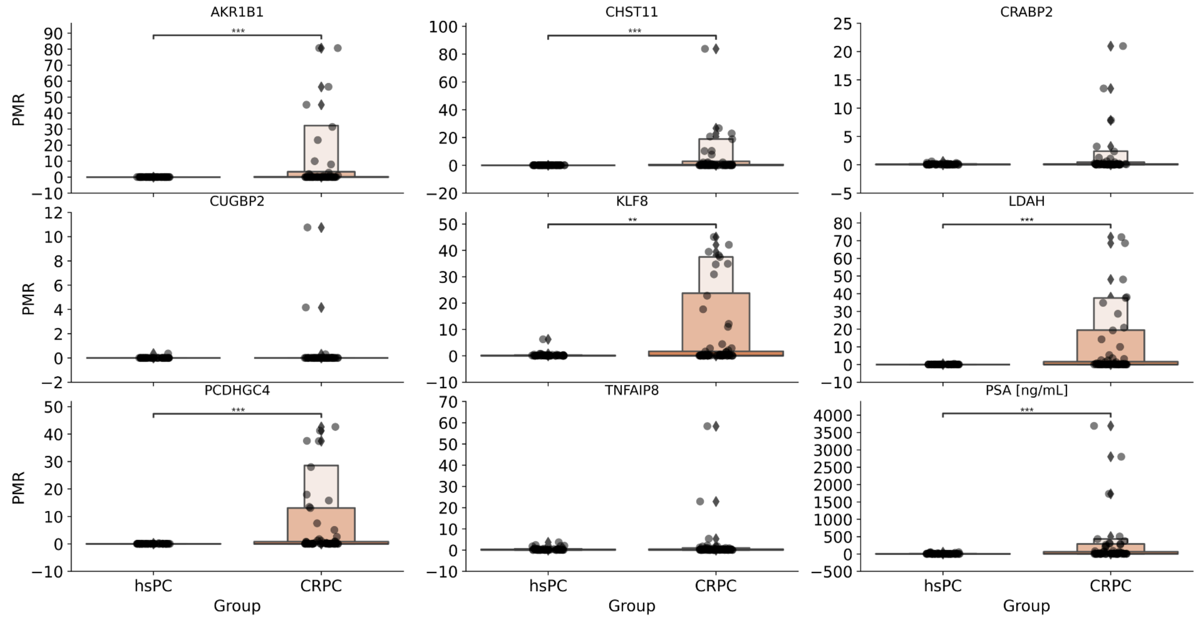

*Figure S1. Box plots depicting the PMR levels of the meth-DNA targets and PSA levels according to castration status. Abbreviations: CRPC - castration-resistant prostate cancer, hsPC - hormone-sensitive prostate cancer, PMR - percentage of methylation ratio, * - p 0.05 - 0.01, ** - p 0.01 - 0.001, *** - p < 0.001*

**Variable**	**hsPC<br>N = 58**	**CRPC<br>N = 64**	**p-Value**
**AKR1B1 [PMR]**	0.00039 (± 0.00198)<br>Range: (1e-06 ; 0.015)	5.38 (± 16.94)<br>Range: (1e-06 ; 80.66)	<0.001
**CHST11 [PMR]**	0.00035 (± 0.0018)<br>Range: (0.0 ; 0.0113)	3.34 (± 11.69)<br>Range: (0.0 ; 83.77)	<0.001
**CRABP2 [PMR]**	0.0813 (± 0.102)<br>Range: (0.000117 ; 0.597)	0.865 (± 3.22)<br>Range: (1.7e-05 ; 20.96)	0.062
**CUGBP2 [PMR]**	0.00637 (± 0.0475)<br>Range: (0.0 ; 0.361)	0.247 (± 1.46)<br>Range: (0.0 ; 10.75)	0.086
**KLF8 [PMR]**	0.238 (± 0.827)<br>Range: (0.00317 ; 6.28)	6.08 (± 12.83)<br>Range: (0.00664 ; 45.07)	0.001
**LDAH [PMR]**	0.0104 (± 0.0596)<br>Range: (4.1e-05 ; 0.452)	6.5 (± 15.71)<br>Range: (4.4e-05 ; 71.99)	<0.001
**PCDHGC4 [PMR]**	0.0118 (± 0.0355)<br>Range: (1e-06 ; 0.254)	4.25 (± 10.52)<br>Range: (1e-06 ; 42.61)	<0.001
**TNFAIP8 [PMR]**	0.397 (± 0.675)<br>Range: (5.5e-05 ; 3.69)	1.66 (± 7.77)<br>Range: (0.0 ; 58.36)	0.737
**PSA [ng/ml]**	6.63 (± 12.29)<br>Range: (0.18 ; 51.2)	189.67 (± 607.63)<br>Range: (0.01 ; 3689.0)	<0.001

Table S3: Tabulated display of PMR levels of the meth-DNA targets and PSA levels according to castration status. Data as mean, standard deviations and ranges.

Meth-DNA PMR and PSA levels according to global PET positivity in all patients

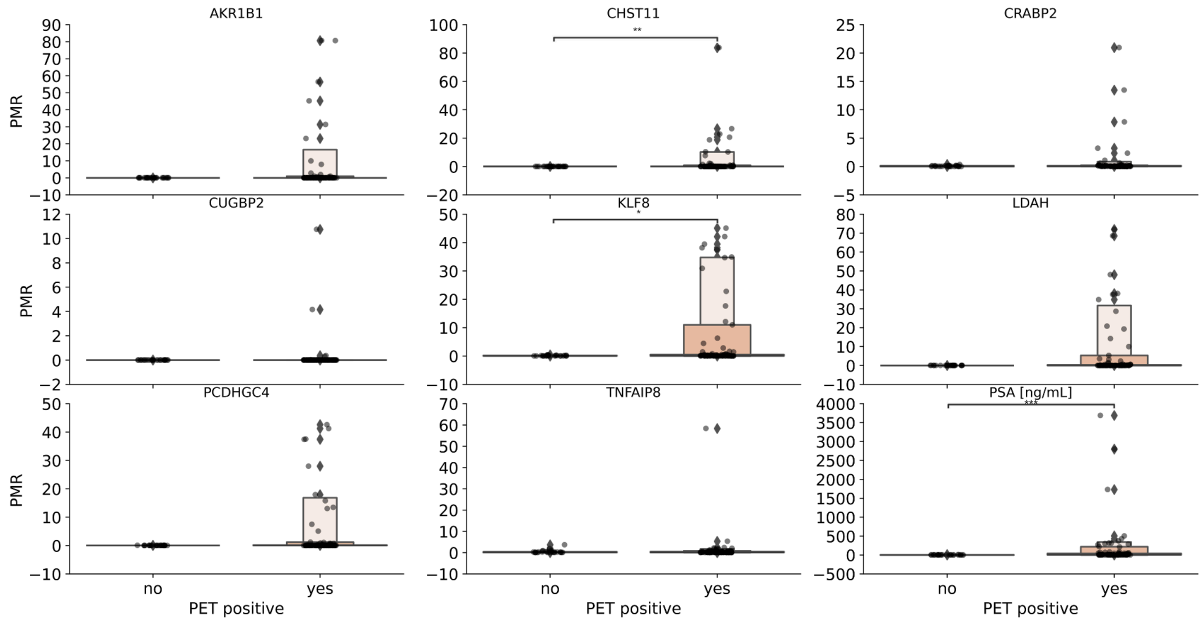

*Figure S2. Box plots depicting the PMR levels of the meth-DNA targets and PSA levels according to global PSMA PET positivity in all patients. Abbreviations: PMR - percentage of methylation ratio, * - p 0.05 - 0.01, ** - p 0.01 - 0.001, *** - p < 0.001*

**Variable**	**Yes**<br>**N = 97**	**No**<br>**N = 25**	**p-Value**
**AKR1B1 [PMR]**	3.55 (± 13.96)<br>Range: (1e-06 ; 80.66)	0.000161 (± 0.000349)<br>Range: (1e-06 ; 0.00171)	0.168
**CHST11 [PMR]**	2.2 (± 9.6)<br>Range: (0.0 ; 83.77)	9.87e-05 (± 0.000476)<br>Range: (0.0 ; 0.00238)	0.007
**CRABP2 [PMR]**	0.6 (± 2.64)<br>Range: (0.000117 ; 20.96)	0.0773 (± 0.0838)<br>Range: (1.7e-05 ; 0.349)	0.238
**CUGBP2 [PMR]**	0.165 (± 1.18)<br>Range: (0.0 ; 10.75)	0.000615 (± 0.00213)<br>Range: (0.0 ; 0.00775)	0.717
**KLF8 [PMR]**	4.12 (± 10.77)<br>Range: (0.00317 ; 45.07)	0.108 (± 0.129)<br>Range: (0.00606 ; 0.427)	0.026
**LDAH [PMR]**	4.29 (± 13.1)<br>Range: (4.1e-05 ; 71.99)	0.00722 (± 0.0126)<br>Range: (4.6e-05 ; 0.0411)	0.063
**PCDHGC4 [PMR]**	2.81 (± 8.76)<br>Range: (1e-06 ; 42.61)	0.0195 (± 0.0249)<br>Range: (1e-06 ; 0.0896)	0.321
**TNFAIP8 [PMR]**	1.21 (± 6.33)<br>Range: (0.0 ; 58.36)	0.469 (± 0.8)<br>Range: (5.5e-05 ; 3.69)	0.477
**PSA [ng/ml]**	128.58 (± 499.69)<br>Range: (0.08 ; 3689.0)	2.05 (± 2.74)<br>Range: (0.01 ; 12.2)	<0.001

Table S4: Tabulated display of PMR levels of the meth-DNA targets and PSA levels according to global PSMA PET positivity in all patients. Data as mean, standard deviations and ranges.

Meth-DNA PMR and PSA levels according to global PET positivity in the hsPC patients

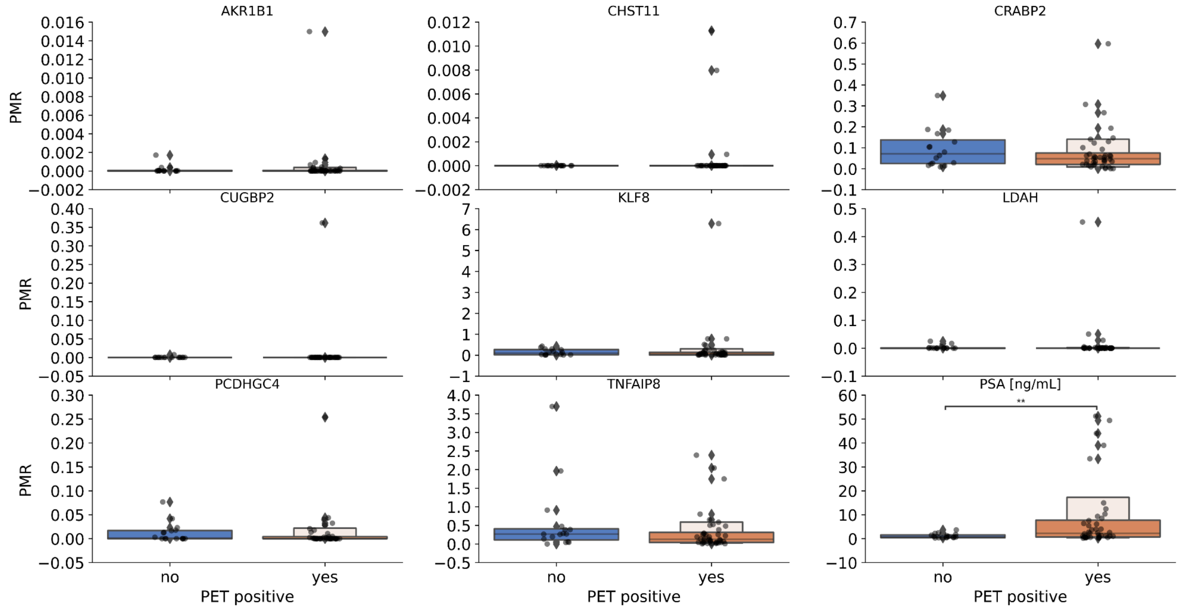

*Figure S3. Box plots depicting the PMR levels of the meth-DNA targets and PSA levels according to global PSMA PET positivity in hsPC patients. Abbreviations: hsPC - hormone-sensitive prostate cancer, PMR - percentage of methylation ratio, * - p 0.05 - 0.01, ** - p 0.01 - 0.001, *** - p < 0.001*

Variable	Yes<br>N = 42	No<br>N = 16	p-Value
**AKR1B1 [PMR]**	0.000474 (± 0.00231)<br>Range: (1e-06 ; 0.015)	0.000169 (± 0.000428)<br>Range: (1e-06 ; 0.00171)	0.601
**CHST11[PMR]**	0.000482 (± 0.00211)<br>Range: (0.0 ; 0.0113)	3.12e-06 (± 8.35e-06)<br>Range: (0.0 ; 3.4e-05)	0.583
**CRABP2 [PMR]**	0.0759 (± 0.106)<br>Range: (0.000117 ; 0.597)	0.0955 (± 0.0916)<br>Range: (0.00809 ; 0.349)	0.293
**CUGBP2 [PMR]**	0.00861 (± 0.0558)<br>Range: (0.0 ; 0.361)	0.000476 (± 0.0019)<br>Range: (0.0 ; 0.0076)	0.532
**KLF8 [PMR]**	0.275 (± 0.968)<br>Range: (0.00317 ; 6.28)	0.142 (± 0.145)<br>Range: (0.00606 ; 0.427)	0.584
**LDAH [PMR]**	0.0131 (± 0.0699)<br>Range: (4.1e-05 ; 0.452)	0.00326 (± 0.00725)<br>Range: (7.2e-05 ; 0.025)	0.554
**PCDHGC4 [PMR]**	0.0114 (± 0.04)<br>Range: (1e-06 ; 0.254)	0.0128 (± 0.0208)<br>Range: (1e-06 ; 0.0768)	0.236
**TNFAIP8 [PMR]**	0.325 (± 0.528)<br>Range: (0.00418 ; 2.39)	0.585 (± 0.957)<br>Range: (5.5e-05 ; 3.69)	0.146
**PSA [ng/ml]**	8.72 (± 13.91)<br>Range: (0.18 ; 51.2)	1.15 (± 0.958)<br>Range: (0.28 ; 3.66)	0.006

Table S5: Tabulated display of PMR levels of the meth-DNA targets and PSA levels according to global PSMA PET positivity in hsPC patients. Data as mean, standard deviations and ranges.

Meth-DNA PMR and PSA levels according to global PET positivity in the CRPC patients

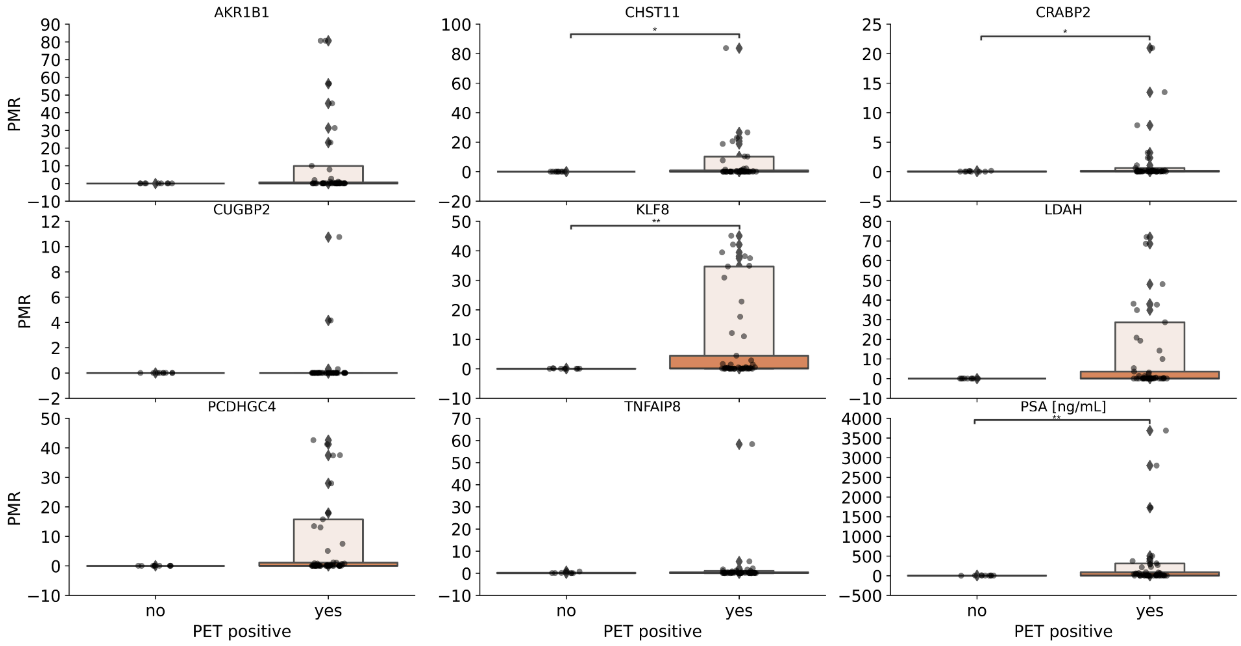

*Figure S4. Box plots depicting the PMR levels of the meth-DNA targets and PSA levels according to global PSMA PET positivity in CRPC patients. Abbreviations: CRPC - castration-resistant prostate cancer, PMR - percentage of methylation ratio, * - p 0.05 - 0.01, ** - p 0.01 - 0.001, *** - p < 0.001*

**Variable**	**Yes N = 55**	**No N = 9**	**p-Value**
**AKR1B1 [PMR]**	6.26 (± 18.14) Range: (1e-06 ; 80.66)	0.000147 (± 0.000152) Range: (1e-06 ; 0.000355)	0.068
**CHST11 [PMR]**	3.89 (± 12.54) Range: (0.0 ; 83.77)	0.000269 (± 0.000792) Range: (0.0 ; 0.00238)	0.011
**CRABP2 [PMR]**	1.0 (± 3.46) Range: (0.00103 ; 20.96)	0.045 (± 0.0594) Range: (1.7e-05 ; 0.147)	0.013
**CUGBP2 [PMR]**	0.288 (± 1.57) Range: (0.0 ; 10.75)	0.000861 (± 0.00258) Range: (0.0 ; 0.00775)	0.524
**KLF8 [PMR]**	7.06 (± 13.61) Range: (0.00664 ; 45.07)	0.0478 (± 0.0621) Range: (0.00718 ; 0.196)	0.002
**LDAH [PMR]**	7.56 (± 16.73) Range: (4.4e-05 ; 71.99)	0.0143 (± 0.0171) Range: (4.6e-05 ; 0.0411)	0.118
**PCDHGC4 [PMR]**	4.94 (± 11.21) Range: (1e-06 ; 42.61)	0.0314 (± 0.0283) Range: (3e-06 ; 0.0896)	0.089
**TNFAIP8 [PMR]**	1.88 (± 8.37) Range: (0.0 ; 58.36)	0.264 (± 0.357) Range: (0.0257 ; 0.965)	0.629
**PSA [ng/ml]**	220.1 (± 651.18) Range: (0.08 ; 3689.0)	3.65 (± 4.05) Range: (0.01 ; 12.2)	0.006

Table S6: Tabulated display of PMR levels of the meth-DNA targets and PSA levels according to global PSMA PET positivity in CRPC patients. Data as mean, standard deviations and ranges.

Meth-DNA PMR and PSA levels according to PSMA PET disease extent in all patients

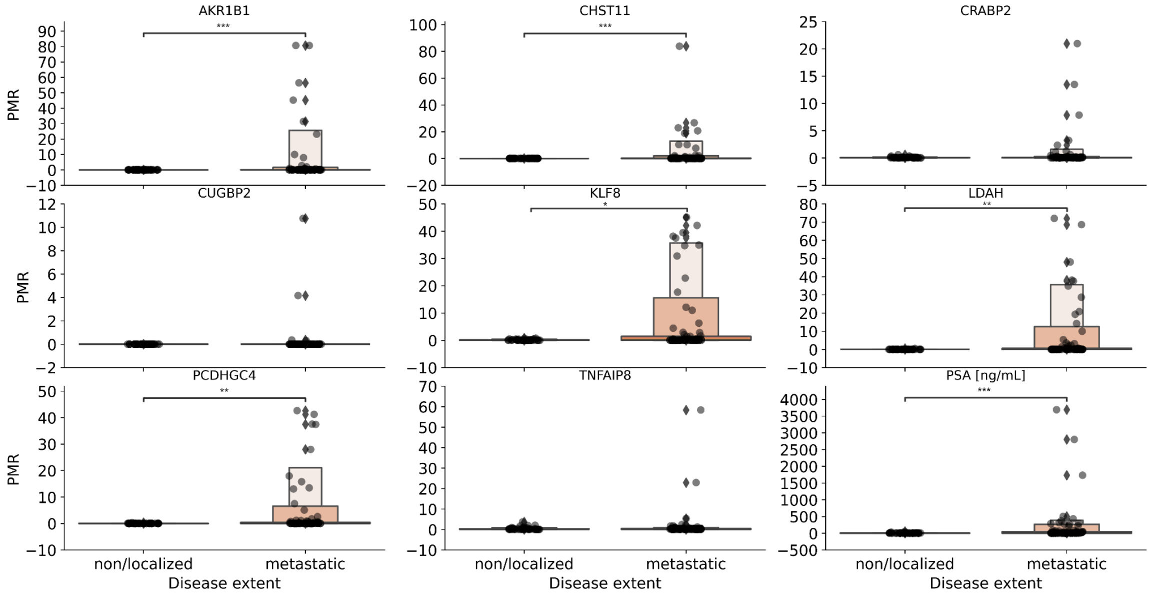

*Figure S5. Box plots depict the PMR levels of the meth-DNA targets and PSA levels according to non-metastatic and metastatic disease on PSMA PET scans in all patients. Abbreviations: PMR - percentage of methylation ratio, * - p 0.05 - 0.01, ** - p 0.01 - 0.001, *** - p <0.001*

Variable	Non/Local<br>N = 46	Metastatic<br>N = 76	p-Value
**AKR1B1 [PMR]**	0.00123 (± 0.00747)<br>Range: (1e-06 ; 0.0507)	4.53 (± 15.65)<br>Range: (1e-06 ; 80.66)	<0.001
**CHST11 [PMR]**	0.00231 (± 0.0151)<br>Range: (0.0 ; 0.103)	2.81 (± 10.78)<br>Range: (0.0 ; 83.77)	<0.001
**CRABP2 [PMR]**	0.0841 (± 0.11)<br>Range: (1.7e-05 ; 0.597)	0.74 (± 2.97)<br>Range: (0.000117 ; 20.96)	0.111
**CUGBP2 [PMR]**	0.000334 (± 0.00158)<br>Range: (0.0 ; 0.00775)	0.211 (± 1.33)<br>Range: (0.0 ; 10.75)	0.154
**KLF8 [PMR]**	0.145 (± 0.195)<br>Range: (0.00606 ; 0.78)	5.21 (± 11.95)<br>Range: (0.00317 ; 45.07)	0.016
**LDAH [PMR]**	0.0173 (± 0.078)<br>Range: (4.1e-05 ; 0.528)	5.47 (± 14.6)<br>Range: (4.4e-05 ; 71.99)	0.007
**PCDHGC4 [PMR]**	0.0209 (± 0.0527)<br>Range: (1e-06 ; 0.336)	3.57 (± 9.77)<br>Range: (1e-06 ; 42.61)	0.002
**TNFAIP8 [PMR]**	0.403 (± 0.695)<br>Range: (5.5e-05 ; 3.69)	1.45 (± 7.14)<br>Range: (0.0 ; 58.36)	0.757
**PSA [ng/mL]**	5.02 (± 9.05)<br>Range: (0.01 ; 51.2)	161.74 (± 560.73)<br>Range: (0.08 ; 3689.0)	<0.001

Table S7: Tabulated display of PMR levels of the meth-DNA targets and PSA levels according to non-metastatic and metastatic disease on PSMA PET scans in all patients. Data as mean, standard deviations and ranges.

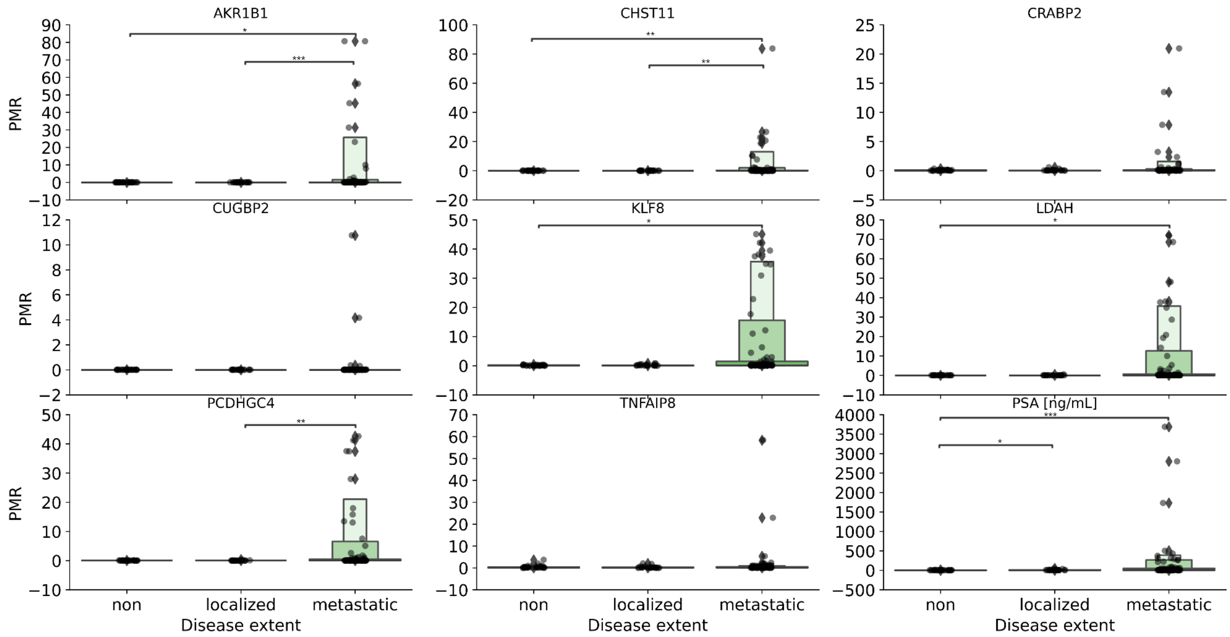

*Figure S6. Box plots depict the PMR levels of the meth-DNA targets and PSA levels according to the disease extent assessed by PSMA PET in all patients. Abbreviations: PMR - percentage of methylation ratio, * - p 0.05 - 0.01, ** - p 0.01 - 0.001, *** - p < 0.001*

**Variable**	**Non N = 25**	**Localized N = 21**	**Metastatic N = 76**	**p-Value**
**AKR1B1 [PMR]**	0.000161 (± 0.000349) Range: (1e-06 ; 0.00171)	0.00251 (± 0.011) Range: (1e-06 ; 0.0507)	4.53 (± 15.65) Range: (1e-06 ; 80.66)	0.001
**CHST11[PMR]**	9.87e-05 (± 0.000476) Range: (0.0 ; 0.00238)	0.00494 (± 0.0224) Range: (0.0 ; 0.103)	2.81 (± 10.78) Range: (0.0 ; 83.77)	<0.001
**CRABP2 [PMR]**	0.0773 (± 0.0838) Range: (1.7e-05 ; 0.349)	0.0922 (± 0.136) Range: (0.000811 ; 0.597)	0.74 (± 2.97) Range: (0.000117 ; 20.96)	0.275
**CUGBP2 [PMR]**	0.000615 (± 0.00213) Range: (0.0 ; 0.00775)	4.76e-08 (± 2.18e-07) Range: (0.0 ; 1e-06)	0.211 (± 1.33) Range: (0.0 ; 10.75)	0.274
**KLF8 [PMR]**	0.108 (± 0.129) Range: (0.00606 ; 0.427)	0.19 (± 0.248) Range: (0.00664 ; 0.78)	5.21 (± 11.95) Range: (0.00317 ; 45.07)	0.037
**LDAH [PMR]**	0.00722 (± 0.0126) Range: (4.6e-05 ; 0.0411)	0.0292 (± 0.115) Range: (4.1e-05 ; 0.528)	5.47 (± 14.6) Range: (4.4e-05 ; 71.99)	0.027
**PCDHGC4 [PMR]**	0.0195 (± 0.0249) Range: (1e-06 ; 0.0896)	0.0227 (± 0.0741) Range: (1e-06 ; 0.336)	3.57 (± 9.77) Range: (1e-06 ; 42.61)	0.004
**TNFAIP8 [PMR]**	0.469 (± 0.8) Range: (5.5e-05 ; 3.69)	0.324 (± 0.556) Range: (0.00345 ; 2.04)	1.45 (± 7.14) Range: (0.0 ; 58.36)	0.458
**PSA [ng/ml]**	2.05 (± 2.74) Range: (0.01 ; 12.2)	8.55 (± 12.3) Range: (0.12 ; 51.2)	161.74 (± 560.73) Range: (0.08 ; 3689.0)	<0.001

Table S8: Tabulated display of PMR levels of the meth-DNA targets and PSA levels according to the disease extent assessed by PSMA PET in all patients. Data as mean, standard deviations and ranges.

AKR1B1 [PMR]	Difference Median	Difference Mean	Mean Difference 95% CI	p-Value
Non VS localized	0.000014	-0.0023	-0.0077 ; 0.003	0.249
Non VS metastatic	-0.000065	-4.53	-12.13 ; 3.07	0.033
localized VS metastatic	-0.000079	-4.53	-12.82 ; 3.77	<0.001
**CHST11 [PMR]**				
Non VS localized	0	-0.0048	-0.016 ; 0.006	0.833
Non VS metastatic	-0.000001	-2.81	-8.05 ; 2.42	0.001
localized VS metastatic	-0.000001	-2.81	-8.52 ; 2.91	0.005
**KLF8 [PMR]**				
Non VS localized	-0.049	-0.082	-0.22 ; 0.057	0.386
Non VS metastatic	-0.12	-5.1	-10.91 ; 0.7	0.014
localized VS metastatic	-0.071	-5.02	-11.36 ; 1.32	0.205
**LDAH [PMR]**				
Non VS localized	-0.000073	-0.022	-0.078 ; 0.034	0.882
Non VS metastatic	-0.00024	-5.46	-12.55 ; 1.63	0.024
localized VS metastatic	-0.00016	-5.44	-13.18 ; 2.3	0.053
**PCDHGC4 [PMR]**				
Non VS localized	0.012	-0.0032	-0.042 ; 0.035	0.194
Non VS metastatic	-0.015	-3.55	-8.3 ; 1.19	0.09
localized VS metastatic	-0.028	-3.55	-8.73 ; 1.63	0.002
**PSA [ng/ml]**				
Non VS localized	-3.65	-6.49	-12.63 ; -0.36	0.019
Non VS metastatic	-7.38	-159.69	-431.92 ; 112.54	<0.001
localized VS metastatic	-3.72	-153.2	-450.58 ; 144.19	0.215

Table S9: Post-hoc Bonferroni adjusted pair-wise PMR value and PSA level differences between PSMA PET disease extent groups in all patients.

Meth-DNA PMR and PSA levels according to PSMA PET disease extent in hsPC patients

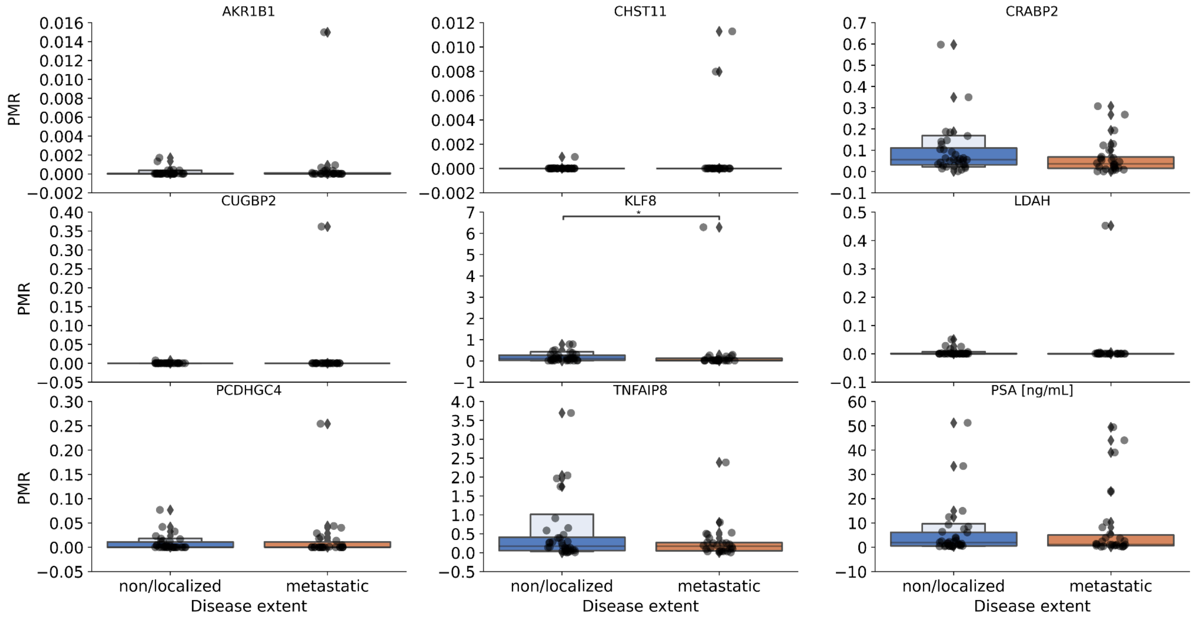

*Figure S7. Box plots depict the PMR levels of the meth-DNA targets and PSA levels according to non-metastatic and metastatic disease on PSMA PET scans in hsPC patients. Abbreviations: hsPC - hormone-sensitive prostate cancer, PMR - percentage of methylation ratio, * - p 0.05 - 0.01, ** - p 0.01 - 0.001, *** - p < 0.001*

Variable	Non/Local<br>N = 32	Metastatic<br>N = 26	p-Value
**AKR1B1**	0.000145 (± 0.000379)<br>Range: (1e-06 ; 0.00171)	0.000691 (± 0.00292)<br>Range: (1e-06 ; 0.015)	0.398
**CHST11**	3.16e-05 (± 0.000167)<br>Range: (0.0 ; 0.000947)	0.000742 (± 0.00266)<br>Range: (0.0 ; 0.0113)	0.459
**CRABP2**	0.094 (± 0.116)<br>Range: (0.000811 ; 0.597)	0.0656 (± 0.0799)<br>Range: (0.000117 ; 0.307)	0.127
**CUGBP2**	0.000238 (± 0.00134)<br>Range: (0.0 ; 0.0076)	0.0139 (± 0.0709)<br>Range: (0.0 ; 0.361)	0.49
**KLF8**	0.18 (± 0.212)<br>Range: (0.00606 ; 0.78)	0.31 (± 1.22)<br>Range: (0.00317 ; 6.28)	0.035
**LDAH**	0.00427 (± 0.011)<br>Range: (4.1e-05 ; 0.0505)	0.0179 (± 0.0886)<br>Range: (5.9e-05 ; 0.452)	0.913
**PCDHGC4**	0.00814 (± 0.0162)<br>Range: (1e-06 ; 0.0768)	0.0163 (± 0.0502)<br>Range: (1e-06 ; 0.254)	0.501
**TNFAIP8**	0.484 (± 0.801)<br>Range: (5.5e-05 ; 3.69)	0.29 (± 0.47)<br>Range: (0.00418 ; 2.39)	0.496
**PSA [ng/mL]**	5.68 (± 10.54)<br>Range: (0.28 ; 51.2)	7.8 (± 14.28)<br>Range: (0.18 ; 49.4)	0.809

Table S10: Tabulated display of PMR levels of the meth-DNA targets and PSA levels according to non-metastatic and metastatic disease on PSMA PET scans in hsPC patients. Data as mean, standard deviations and ranges.

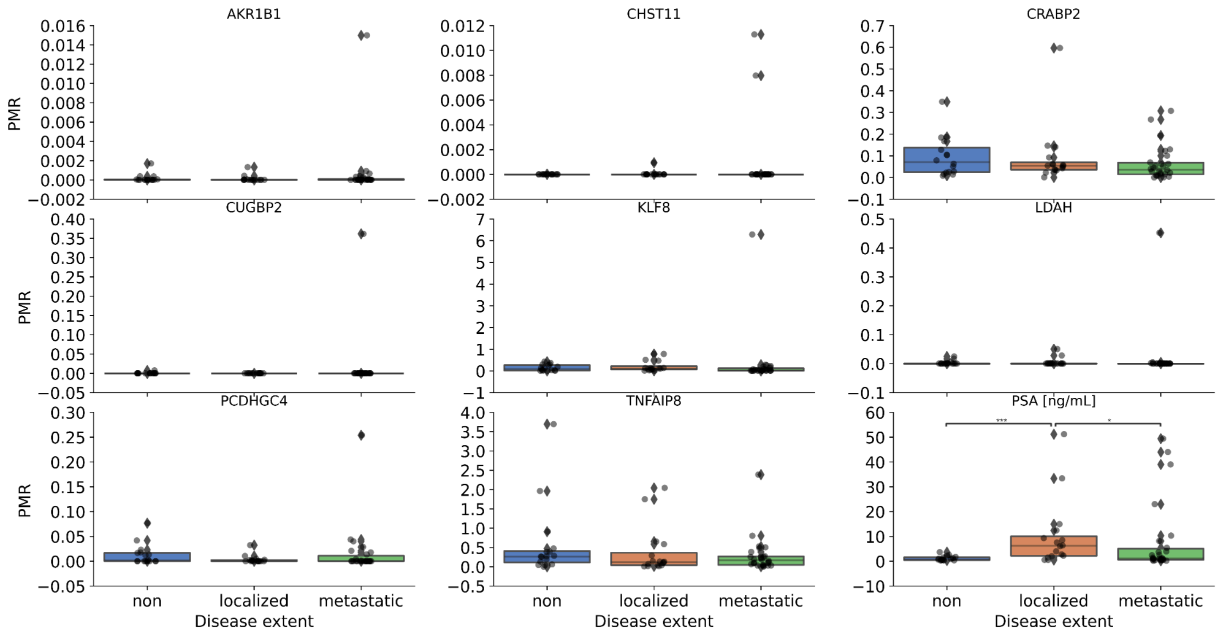

*Figure S8. Box plots depict the PMR levels of the meth-DNA targets according to the disease extent assessed by PSMA PET in hsPC patients. Abbreviations: hsPC - hormone-sensitive prostate cancer, PMR - percentage of methylation ratio, * - p 0.05 - 0.01, ** - p 0.01-0.001, *** - p <0.001*

Variable	Non N = 16	localized N = 16	metastatic N = 26	p-Value
**AKR1B1 [PMR]**	0.000169 (± 0.000428) Range: (1e-06 ; 0.00171)	0.000121 (± 0.000337) Range: (1e-06 ; 0.00132)	0.000691 (± 0.00292) Range: (1e-06 ; 0.015)	0.334
**CHST11 [PMR]**	3.13e-06 (± 8.35e-06) Range: (0.0 ; 3.4e-05)	6e-05 (± 0.000237) Range: (0.0 ; 0.000947)	0.000742 (± 0.00266) Range: (0.0 ; 0.0113)	0.745
**CRABP2 [PMR]**	0.0955 (± 0.0916) Range: (0.00809 ; 0.349)	0.0926 (± 0.14) Range: (0.000811 ; 0.597)	0.0656 (± 0.0799) Range: (0.000117 ; 0.307)	0.3
**CUGBP2 [PMR]**	0.000476 (± 0.0019) Range: (0.0 ; 0.0076)	0.0 (± 0.0) Range: (0.0 ; 0.0)	0.0139 (± 0.0709) Range: (0.0 ; 0.361)	0.358
**KLF8 [PMR]**	0.142 (± 0.145) Range: (0.00606 ; 0.427)	0.217 (± 0.263) Range: (0.00737 ; 0.78)	0.31 (± 1.22) Range: (0.00317 ; 6.28)	0.082
**LDAH [PMR]**	0.00326 (± 0.00725) Range: (7.2e-05 ; 0.025)	0.00529 (± 0.0139) Range: (4.1e-05 ; 0.0505)	0.0179 (± 0.0886) Range: (5.9e-05 ; 0.452)	0.721
**PCDHGC4 [PMR]**	0.0128 (± 0.0208) Range: (1e-06 ; 0.0768)	0.0035 (± 0.00819) Range: (1e-06 ; 0.0324)	0.0163 (± 0.0502) Range: (1e-06 ; 0.254)	0.491
**TNFAIP8 [PMR]**	0.585 (± 0.957) Range: (5.5e-05 ; 3.69)	0.383 (± 0.623) Range: (0.00781 ; 2.04)	0.29 (± 0.47) Range: (0.00418 ; 2.39)	0.34
**PSA [ng/ml]**	1.15 (± 0.958) Range: (0.28 ; 3.66)	10.2 (± 13.61) Range: (0.52 ; 51.2)	7.8 (± 14.28) Range: (0.18 ; 49.4)	0.002

Table S11: Tabulated display of PMR levels of the meth-DNA targets and PSA levels according to the disease extent assessed by PSMA PET in hsPC patients. Data as mean, standard deviations and ranges.

PSA [ng/ml]	Difference Median	Difference Mean	Mean Difference 95% CI	p-Value
Non VS localized	-5.45	-9.05	-17.43 ; -0.68	<0.001
Non VS metastatic	-0.37	-6.65	-15.45 ; 2.15	0.081
localized VS metastatic	5.08	2.41	-8.47 ; 13.28	0.031

Table S12: Post-hoc Bonferroni adjusted PSA level differences between PSMA PET disease extent groups in all patients

Meth-DNA PMR and PSA levels according to PSMA PET disease extent in CRPC patients

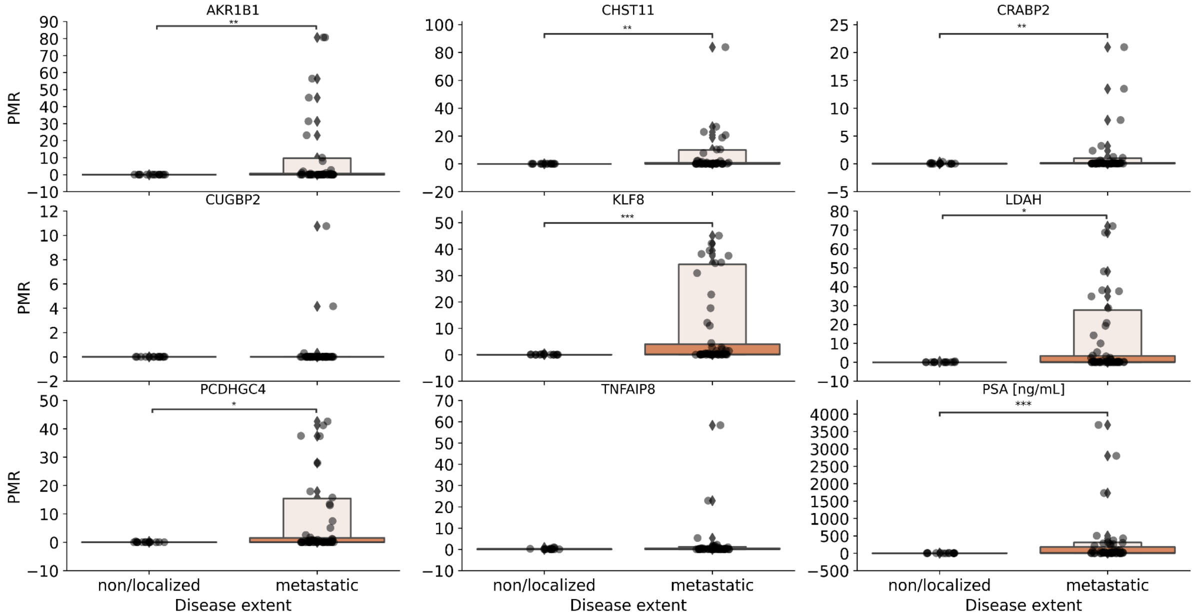

*Figure S9. Box plots depict the PMR levels of the meth-DNA targets and PSA levels according to non-metastatic and metastatic disease on PSMA PET scans in CRPC patients. Abbreviations: CRPC - castration-resistant prostate cancer, PMR - percentage of methylation ratio, * - p 0.05 - 0.01, ** - p 0.01 - 0.001, *** - p < 0.001*

**Variable**	**Non/Local N = 14**	**Metastatic N = 50**	**p-Value**
**AKR1B1**	0.00372 (± 0.0135) Range: (1e-06 ; 0.0507)	6.88 (± 18.93) Range: (1e-06 ; 80.66)	0.007
**CHST11**	0.00751 (± 0.0274) Range: (0.0 ; 0.103)	4.27 (± 13.1) Range: (0.0 ; 83.77)	0.001
**CRABP2**	0.0614 (± 0.0936) Range: (1.7e-05 ; 0.342)	1.09 (± 3.62) Range: (0.00103 ; 20.96)	0.004
**CUGBP2**	0.000554 (± 0.00207) Range: (0.0 ; 0.00775)	0.318 (± 1.65) Range: (0.0 ; 10.75)	0.526
**KLF8**	0.067 (± 0.119) Range: (0.00664 ; 0.442)	7.76 (± 14.09) Range: (0.0074 ; 45.07)	<0.001
**LDAH**	0.047 (± 0.139) Range: (4.6e-05 ; 0.528)	8.3 (± 17.38) Range: (4.4e-05 ; 71.99)	0.026
**PCDHGC4**	0.0502 (± 0.0874) Range: (2e-06 ; 0.336)	5.42 (± 11.65) Range: (1e-06 ; 42.61)	0.018
**TNFAIP8**	0.219 (± 0.305) Range: (0.00345 ; 0.965)	2.06 (± 8.76) Range: (0.0 ; 58.36)	0.22
**PSA [ng/mL]**	3.5 (± 3.87) Range: (0.01 ; 12.2)	241.79 (± 679.73) Range: (0.08 ; 3689.0)	<0.001

Table S13: Tabulated display of PMR levels of the meth-DNA targets and PSA levels according to the disease extent assessed by PSMA PET in CRPC patients. Data as mean, standard deviations and ranges.

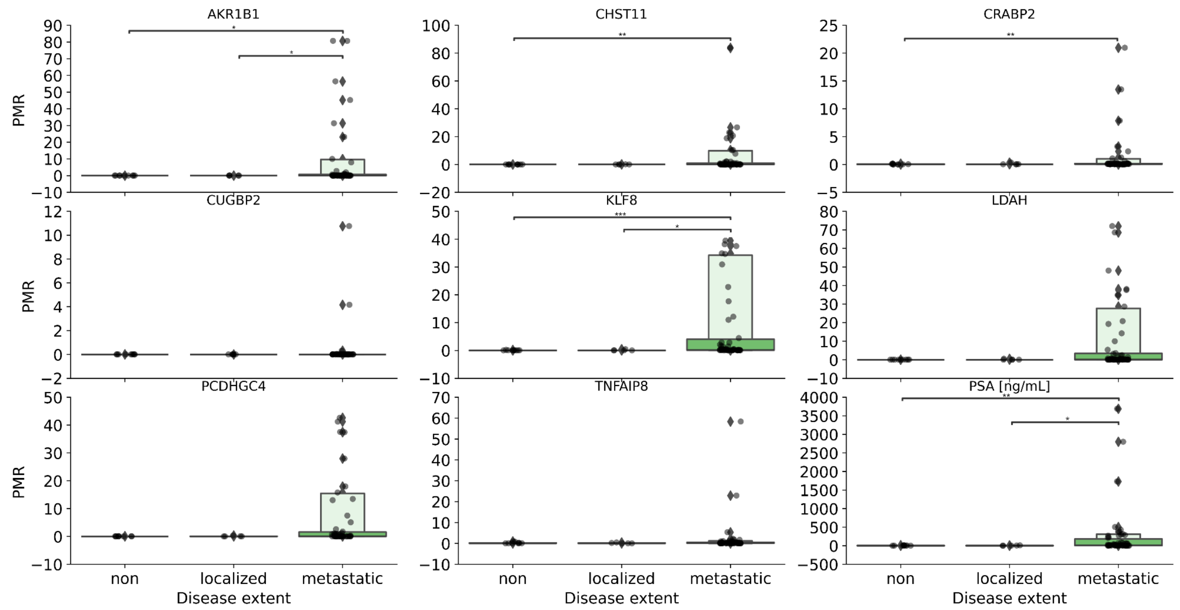

*Figure S10. Box plots depict the PMR levels of the meth-DNA targets and PSA levels according to the disease extent assessed by PSMA PET in CRPC patients. Abbreviations: CRPC - castration-resistant prostate cancer, PMR - percentage of methylation ratio, * - p 0.05 - 0.01, ** - p 0.01 - 0.001, *** - p < 0.001*

Variable	Non<br>N = 9	Localized<br>N = 5	Metastatic<br>N = 50	p-Value
**AKR1B1 [PMR]**	0.000147 (± 0.000152)<br>Range: (1e-06 ; 0.000355)	0.0101 (± 0.0227)<br>Range: (2e-06 ; 0.0507)	6.88 (± 18.93)<br>Range: (1e-06 ; 80.66)	0.022
**CHST11 [PMR]**	0.000269 (± 0.000792)<br>Range: (0.0 ; 0.00238)	0.0205 (± 0.0459)<br>Range: (0.0 ; 0.103)	4.27 (± 13.1)<br>Range: (0.0 ; 83.77)	0.006
**CRABP2 [PMR]**	0.045 (± 0.0594)<br>Range: (1.7e-05 ; 0.147)	0.0909 (± 0.141)<br>Range: (0.0182 ; 0.342)	1.09 (± 3.62)<br>Range: (0.00103 ; 20.96)	0.014
**CUGBP2 [PMR]**	0.000861 (± 0.00258)<br>Range: (0.0 ; 0.00775)	2e-07 (± 4.47e-07)<br>Range: (0.0 ; 1e-06)	0.318 (± 1.65)<br>Range: (0.0 ; 10.75)	0.787
**KLF8 [PMR]**	0.0478 (± 0.0621)<br>Range: (0.00718 ; 0.196)	0.101 (± 0.191)<br>Range: (0.00664 ; 0.442)	7.76 (± 14.09)<br>Range: (0.0074 ; 45.07)	<0.001
**LDAH [PMR]**	0.0143 (± 0.0171)<br>Range: (4.6e-05 ; 0.0411)	0.106 (± 0.236)<br>Range: (0.000116 ; 0.528)	8.3 (± 17.38)<br>Range: (4.4e-05 ; 71.99)	0.079
**PCDHGC4 [PMR]**	0.0314 (± 0.0283)<br>Range: (3e-06 ; 0.0896)	0.0841 (± 0.145)<br>Range: (2e-06 ; 0.336)	5.42 (± 11.65)<br>Range: (1e-06 ; 42.61)	0.058
**TNFAIP8 [PMR]**	0.264 (± 0.357)<br>Range: (0.0257 ; 0.965)	0.137 (± 0.181)<br>Range: (0.00345 ; 0.437)	2.06 (± 8.76)<br>Range: (0.0 ; 58.36)	0.366
**PSA [ng/mL]**	3.65 (± 4.05)<br>Range: (0.01 ; 12.2)	3.23 (± 3.96)<br>Range: (0.12 ; 9.53)	241.79 (± 679.73)<br>Range: (0.08 ; 3689.0)	0.001

Table S14: Tabulated display of PMR levels of the meth-DNA targets and PSA levels according to the disease extent assessed by PSMA PET in CRPC patients. Data as mean, standard deviations and ranges.

**AKR1B1 [PMR]**	Difference Median	Difference Mean	Mean Difference 95% CI	p-Value
Non VS Localized	0.00013	-0.01	-0.028 ; 0.0082	0.697
Non VS Metastatic	-0.0017	-6.88	-22.4 ; 8.64	0.039
Localized VS Metastatic	-0.0018	-6.87	-27.77 ; 14.02	0.04
**CHST11 [PMR]**				
Non VS Localized	0	-0.02	-0.057 ; 0.017	0.852

Non VS Metastatic	-0.0043	-4.27	-15.01 ; 6.46	0.006
Localized VS Metastatic	-0.0043	-4.25	-18.71 ; 10.2	0.055
**CRABP2 [PMR]**				
Non VS Localized	-0.015	-0.046	-0.18 ; 0.087	0.638
Non VS Metastatic	-0.07	-1.05	-4.01 ; 1.92	0.008
Localized VS Metastatic	-0.055	-1	-5 ; 3	0.135
**KLF8 [PMR]**				
Non VS Localized	0.0048	-0.054	-0.22 ; 0.12	0.928
Non VS Metastatic	-0.24	-7.71	-19.26 ; 3.84	<0.001
Localized VS Metastatic	-0.25	-7.66	-23.21 ; 7.9	0.013
**PSA [ng/ml]**				
Non VS localized	1.81	0.42	-5.36 ; 6.19	0.94
Non VS metastatic	-12.68	-238.14	-795.38 ; 319.1	0.003
localized VS metastatic	-14.48	-238.56	-988.85 ; 511.73	0.016

Table S15: Post-hoc Bonferroni adjusted pair-wise PMR value differences and PSMA levels between PSMA PET disease extent groups in CRPC patients

Target	Group	ROC Value	Lower CI	Upper CI	Significance
AKR1B1	ALL	0.59	0.479	0.701	0
CHST11	ALL	0.671	0.566	0.776	1
CRABP2	ALL	0.577	0.44	0.714	0
CUGBP2	ALL	0.525	0.451	0.598	0
KLF8	ALL	0.645	0.534	0.757	1
LDAH	ALL	0.621	0.503	0.739	0
PCDHGC4	ALL	0.565	0.456	0.673	0
PSA	ALL	0.77	0.683	0.857	1
TNFAIP8	ALL	0.546	0.42	0.673	0
AKR1B1	hsPC	0.545	0.379	0.712	0
CHST11	hsPC	0.545	0.375	0.716	0
CRABP2	hsPC	0.591	0.415	0.766	0
CUGBP2	hsPC	0.527	0.434	0.619	0
KLF8	hsPC	0.548	0.374	0.721	0
LDAH	hsPC	0.551	0.369	0.734	0
PCDHGC4	hsPC	0.602	0.433	0.771	0
PSA	hsPC	0.737	0.609	0.866	1
TNFAIP8	hsPC	0.625	0.458	0.792	0
AKR1B1	CRPC	0.692	0.534	0.85	0
CHST11	CRPC	0.766	0.633	0.898	1
CRABP2	CRPC	0.762	0.556	0.967	1
CUGBP2	CRPC	0.564	0.442	0.685	0

KLF8	CRPC	0.824	0.708	0.941	1
LDAH	CRPC	0.665	0.513	0.817	0
PCDHGC4	CRPC	0.679	0.544	0.813	0
PSA	CRPC	0.786	0.652	0.92	1
TNFAIP8	CRPC	0.552	0.358	0.745	0

Table S16: AUC [95% CI] values of ROC analysis to distinguish between global PET positivity. Abbreviations: hsPC - hormone-sensitive prostate cancer, CRPC - castration-resistant prostate cancer

Target	Group	ROC Value	Lower CI	Upper CI	Significance
AKR1B1	ALL	0.687	0.594	0.78	1
CHST11	ALL	0.705	0.618	0.793	1
CRABP2	ALL	0.586	0.482	0.691	0
CUGBP2	ALL	0.559	0.499	0.619	0
KLF8	ALL	0.631	0.533	0.729	1
LDAH	ALL	0.645	0.548	0.742	1
PCDHGC4	ALL	0.664	0.57	0.759	1
TNFAIP8	ALL	0.517	0.41	0.624	0
PSA	ALL	0.672	0.577	0.768	1
AKR1B1	hsPC	0.566	0.416	0.715	0
CHST11	hsPC	0.555	0.41	0.699	0
CRABP2	hsPC	0.618	0.468	0.767	0
CUGBP2	hsPC	0.526	0.451	0.602	0
KLF8	hsPC	0.662	0.519	0.806	1
LDAH	hsPC	0.509	0.357	0.661	0
PCDHGC4	hsPC	0.552	0.397	0.708	0
TNFAIP8	hsPC	0.553	0.402	0.703	0
PSA	hsPC	0.543	0.39	0.697	0
AKR1B1	CRPC	0.74	0.609	0.871	1
CHST11	CRPC	0.779	0.662	0.895	1
CRABP2	CRPC	0.753	0.59	0.915	1
CUGBP2	CRPC	0.558	0.452	0.663	0
KLF8	CRPC	0.85	0.743	0.957	1
LDAH	CRPC	0.697	0.567	0.827	1
PCDHGC4	CRPC	0.709	0.579	0.839	0
TNFAIP8	CRPC	0.609	0.446	0.772	0
PSA	CRPC	0.812	0.703	0.922	1

Table S17: AUC [95% CI] values of ROC analysis to distinguish between non-metastatic and metastatic disease on PSMA PET imaging. Abbreviations: hsPC - hormone-sensitive prostate cancer, CRPC - castration-resistant prostate cancer

Meth-DNA PMR and PSA levels correlation with PSMA-TV according to castration group

Variable	Group	Correlation Coefficient	p-Value	Adjusted p-Value
**AKR1B1 [PMR]**	All	0.4	<0.001	<0.001
**CHST11 [PMR]**	All	0.44	<0.001	<0.001
**CRABP2 [PMR]**	All	0.23	0.012	0.33
**CUGBP2 [PMR]**	All	0.11	0.24	1
**KLF8 [PMR]**	All	0.37	<0.001	<0.001
**LDAH [PMR]**	All	0.44	<0.001	<0.001
**PCDHGC4 [PMR]**	All	0.46	<0.001	<0.001
**PSA [ng/mL]**	All	0.71	<0.001	<0.001
**TNFAIP8 [PMR]**	All	-0.045	0.626	1
**AKR1B1 [PMR]**	hsPC	-0.1	0.438	1
**CHST11 [PMR]**	hsPC	-0.079	0.557	1
**CRABP2 [PMR]**	hsPC	-0.24	0.069	1
**CUGBP2 [PMR]**	hsPC	-0.065	0.625	1
**KLF8 [PMR]**	hsPC	-0.24	0.071	1
**LDAH [PMR]**	hsPC	0.046	0.733	1
**PCDHGC4 [PMR]**	hsPC	-0.025	0.85	1
**PSA [ng/mL]**	hsPC	0.58	<0.001	<0.001
**TNFAIP8 [PMR]**	hsPC	-0.25	0.06	1
**AKR1B1 [PMR]**	CRPC	0.56	<0.001	<0.001
**CHST11 [PMR]**	CRPC	0.58	<0.001	<0.001
**CRABP2 [PMR]**	CRPC	0.44	<0.001	0.002
**CUGBP2 [PMR]**	CRPC	0.072	0.578	1
**KLF8 [PMR]**	CRPC	0.62	<0.001	<0.001
**LDAH [PMR]**	CRPC	0.57	<0.001	<0.001
**PCDHGC4 [PMR]**	CRPC	0.51	<0.001	<0.001
**PSA [ng/mL]**	CRPC	0.76	<0.001	<0.001
**TNFAIP8 [PMR]**	CRPC	0.081	0.527	1

Table S18: Correlations between Meth-DNA PMR and PSA levels with PSMA-TV according to castration status. p-values as non- and Bonferroni-adjusted.

Stratifying median cut-offs used in the survival analysis

Variable	Overall cohort median cut-off	CRPC cohort median cut-off
AKR1B1 [PMR]	0.000157	0.000254
CHST11 [PMR]	0.00000113	0.000005511
CRABP2 [PMR]	0.060249378	0.073574344
CUGBP2 [PMR]	0.0000000000000085	0.0000000000000085
KLF8 [PMR]	0.103984828	0.185858731
LDAH [PMR]	0.000264643	0.027337261
PCDHGC4 [PMR]	0.011765058	0.058613206
TNFAIP8 [PMR]	0.147069081	0.134998039
PSA [ng/mL]	3.96	9.65
PSMA [cm^3]	4.03	17.52

Table S20: Cut-offs used to stratify high and low groups based on the median variable values of the overall cohort and CRPC group

Univariate log-rank test comparison of survival distributions

Variable [overall median]	p-Value	Variable [CRPC median]	p-Value
AKR1B1	0.00701	AKR1B1	p<0.0001
CHST11	0.000287	CHST11	p<0.0001
CRABP2	0.0322	CRABP2	0.00704
CUGBP2	0.681	CUGBP2	0.88
KLF8	0.000144	KLF8	p<0.0001
LDAH	0.000656	LDAH	p<0.0001
PCDHGC4	0.00927	PCDHGC4	p<0.0001
PSA	0.000478	PSA	p<0.0001
PSMA-TV	0.00034	PSMA-TV	p<0.0001
TNFAIP8	0.511	TNFAIP8	0.674

Table S20: Univariate log-rank tested survival difference results between groups of high and low methylation DNA marker PMR values, PSA and PSMA-TV levels. Group stratification by median of the overall cohort (left) and by the median of the CRPC cohort (right)

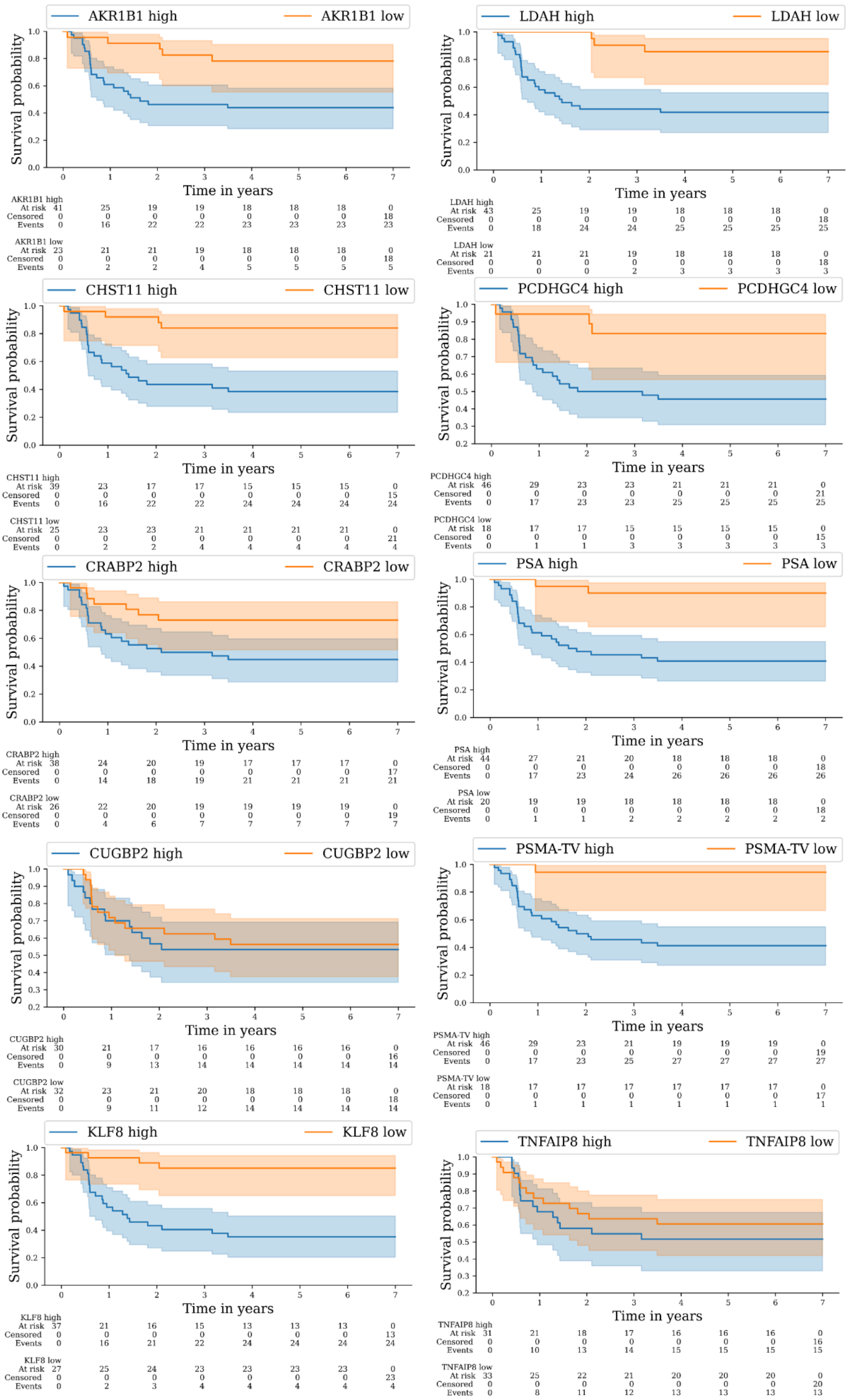
AKR1B1 high
AKR1B1 low
Survival probability
Time in years
AKR1B1 high
At risk 41 25 19 19 18 18 18 0
Censored 0 0 0 0 0 0 0 18
Events 0 16 22 22 23 23 23 23
AKR1B1 low
At risk 23 21 21 19 18 18 18 0
Censored 0 0 0 0 0 0 0 18
Events 0 2 2 4 5 5 5 5
LDAH high
LDAH low
Survival probability
Time in years
LDAH high
At risk 43 25 19 19 18 18 18 0
Censored 0 0 0 0 0 0 0 18
Events 0 18 24 24 25 25 25 25
LDAH low
At risk 21 21 21 19 18 18 18 0
Censored 0 0 0 0 0 0 0 18
Events 0 0 0 2 3 3 3 3
CHST11 high
CHST11 low
Survival probability
Time in years
CHST11 high
At risk 39 23 17 17 15 15 15 0
Censored 0 0 0 0 0 0 0 15
Events 0 16 22 22 24 24 24 24
CHST11 low
At risk 25 23 23 21 21 21 21 0
Censored 0 0 0 0 0 0 0 21
Events 0 2 2 4 4 4 4 4
PCDHGC4 high
PCDHGC4 low
Survival probability
Time in years
PCDHGC4 high
At risk 46 29 23 23 21 21 21 0
Censored 0 0 0 0 0 0 0 21
Events 0 17 23 23 25 25 25 25
PCDHGC4 low
At risk 18 17 17 15 15 15 15 0
Censored 0 0 0 0 0 0 0 15
Events 0 1 1 3 3 3 3 3
CRABP2 high
CRABP2 low
Survival probability
Time in years
CRABP2 high
At risk 38 24 20 19 17 17 17 0
Censored 0 0 0 0 0 0 0 17
Events 0 14 18 19 21 21 21 21
CRABP2 low
At risk 26 22 20 19 19 19 19 0
Censored 0 0 0 0 0 0 0 19
Events 0 4 6 7 7 7 7 7
PSA high
PSA low
Survival probability
Time in years
PSA high
At risk 44 27 21 20 18 18 18 0
Censored 0 0 0 0 0 0 0 18
Events 0 17 23 24 26 26 26 26
PSA low
At risk 20 19 19 18 18 18 18 0
Censored 0 0 0 0 0 0 0 18
Events 0 1 1 2 2 2 2 2
CUGBP2 high
CUGBP2 low
Survival probability
Time in years
CUGBP2 high
At risk 30 21 17 16 16 16 16 0
Censored 0 0 0 0 0 0 0 16
Events 0 9 13 14 14 14 14 14
CUGBP2 low
At risk 32 23 21 20 18 18 18 0
Censored 0 0 0 0 0 0 0 18
Events 0 9 11 12 14 14 14 14
PSMA-TV high
PSMA-TV low
Survival probability
Time in years
PSMA-TV high
At risk 46 29 23 21 19 19 19 0
Censored 0 0 0 0 0 0 0 19
Events 0 17 23 25 27 27 27 27
PSMA-TV low
At risk 18 17 17 17 17 17 17 0
Censored 0 0 0 0 0 0 0 17
Events 0 1 1 1 1 1 1 1
KLF8 high
KLF8 low
Survival probability
Time in years
KLF8 high
At risk 37 21 16 15 13 13 13 0
Censored 0 0 0 0 0 0 0 13
Events 0 16 21 22 24 24 24 24
KLF8 low
At risk 27 25 24 23 23 23 23 0
Censored 0 0 0 0 0 0 0 23
Events 0 2 3 4 4 4 4 4
TNFAIP8 high
TNFAIP8 low
Survival probability
Time in years
TNFAIP8 high
At risk 31 21 18 17 16 16 16 0
Censored 0 0 0 0 0 0 0 16
Events 0 10 13 14 15 15 15 15
TNFAIP8 low
At risk 33 25 22 21 20 20 20 0
Censored 0 0 0 0 0 0 0 20
Events 0 8 11 12 13 13 13 13

Figure S11. Kaplan Meier curves depicting the survival in high and low groups of the DNA methylation markers, PSA and PSMA-TV levels using their respective median values of the overall cohort as stratifying cut-off.

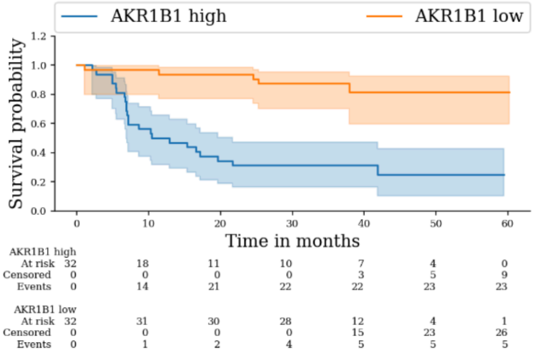
AKR1B1 high
AKR1B1 low
Survival probability
Time in months
AKR1B1 high
At risk 32 18 11 10 7 4 0
Censored 0 0 0 0 3 5 9
Events 0 14 21 22 22 23 23
AKR1B1 low
At risk 32 31 30 28 12 4 1
Censored 0 0 0 0 15 23 26
Events 0 1 2 4 5 5 5

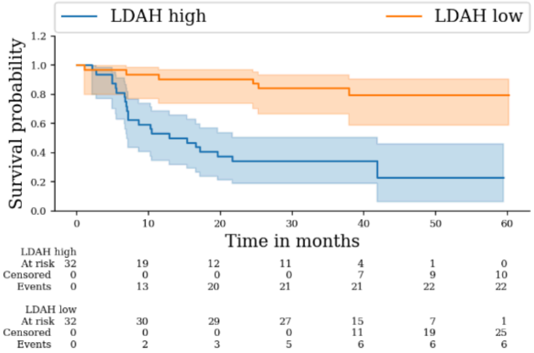
LDAH high
LDAH low
Survival probability
Time in months
LDAH high
At risk 32 19 12 11 4 1 0
Censored 0 0 0 0 7 9 10
Events 0 13 20 21 21 22 22
LDAH low
At risk 32 30 29 27 15 7 1
Censored 0 0 0 0 11 19 25
Events 0 2 3 5 6 6 6

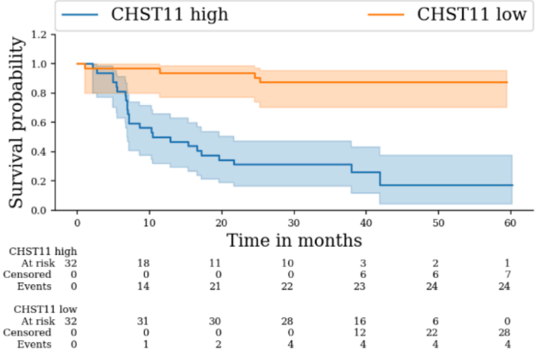
CHST11 high
CHST11 low
Survival probability
Time in months
CHST11 high
At risk 32 18 11 10 3 2 1
Censored 0 0 0 0 6 6 7
Events 0 14 21 22 23 24 24
CHST11 low
At risk 32 31 30 28 16 6 0
Censored 0 0 0 0 12 22 28
Events 0 1 2 4 4 4 4

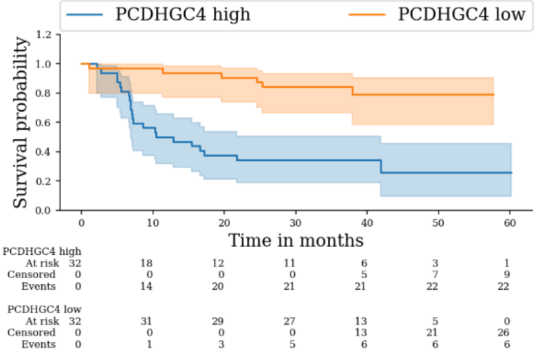
PCDHGC4 high
PCDHGC4 low
Survival probability
Time in months
PCDHGC4 high
At risk 32 18 12 11 6 3 1
Censored 0 0 0 0 5 7 9
Events 0 14 20 21 21 22 22
PCDHGC4 low
At risk 32 31 29 27 13 5 0
Censored 0 0 0 0 13 21 26
Events 0 1 3 5 6 6 6

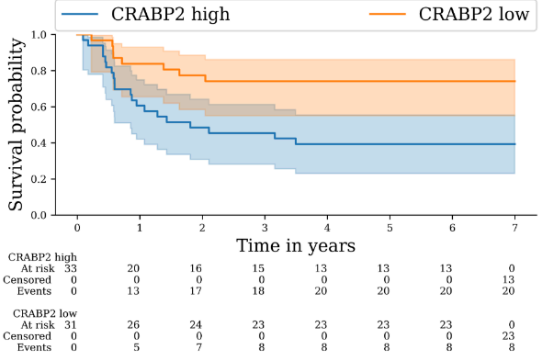
CRABP2 high
CRABP2 low
Survival probability
Time in years
CRABP2 high
At risk 33 20 16 15 13 13 13 0
Censored 0 0 0 0 0 0 0 13
Events 0 13 17 18 20 20 20 20
CRABP2 low
At risk 31 26 24 23 23 23 23 0
Censored 0 0 0 0 0 0 0 23
Events 0 5 7 8 8 8 8 8

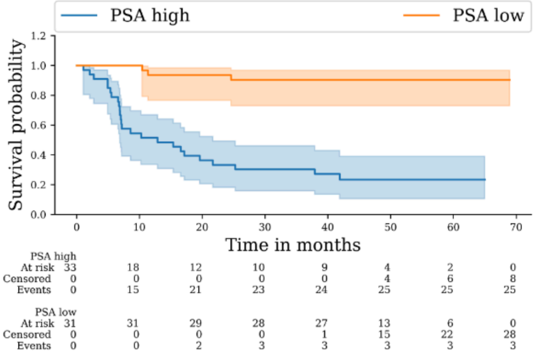
PSA high
PSA low
Survival probability
Time in months
PSA high
At risk 33 18 12 10 9 4 2 0
Censored 0 0 0 0 0 4 6 8
Events 0 15 21 23 24 25 25 25
PSA low
At risk 31 31 29 28 27 13 6 0
Censored 0 0 0 0 1 15 22 28
Events 0 0 2 3 3 3 3 3

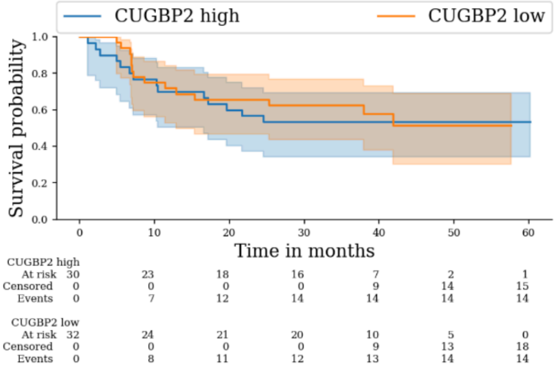
CUGBP2 high
CUGBP2 low
Survival probability
Time in months
CUGBP2 high
At risk 30 23 18 16 7 2 1
Censored 0 0 0 0 9 14 15
Events 0 7 12 14 14 14 14
CUGBP2 low
At risk 32 24 21 20 10 5 0
Censored 0 0 0 0 9 13 18
Events 0 8 11 12 13 14 14

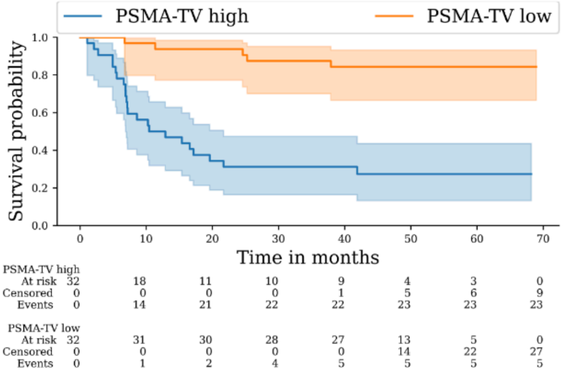
PSMA-TV high
PSMA-TV low
Survival probability
Time in months
PSMA-TV high
At risk 32 18 11 10 9 4 3 0
Censored 0 0 0 0 1 5 6 9
Events 0 14 21 22 22 23 23 23
PSMA-TV low
At risk 32 31 30 28 27 13 5 0
Censored 0 0 0 0 0 14 22 27
Events 0 1 2 4 5 5 5 5

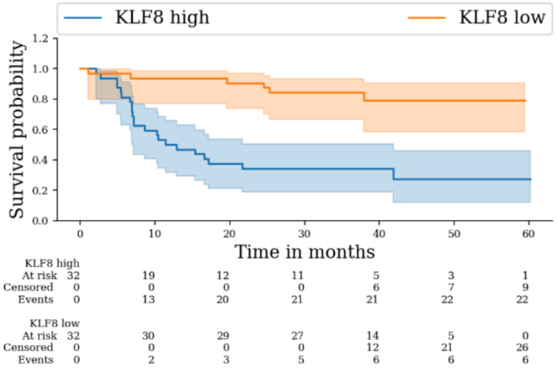
KLF8 high
KLF8 low
Survival probability
Time in months
KLF8 high
At risk 32 19 12 11 5 3 1
Censored 0 0 0 0 6 7 9
Events 0 13 20 21 21 22 22
KLF8 low
At risk 32 30 29 27 14 5 0
Censored 0 0 0 0 12 21 26
Events 0 2 3 5 6 6 6

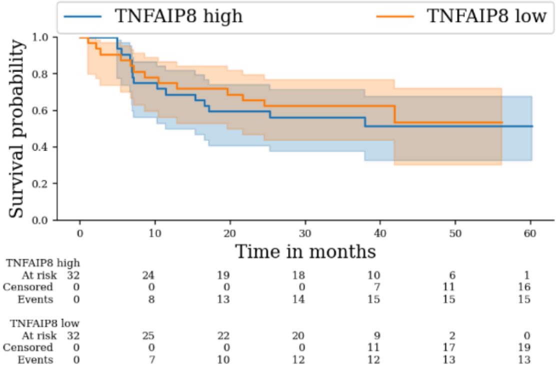
TNFAIP8 high
TNFAIP8 low
Survival probability
Time in months
TNFAIP8 high
At risk 32 24 19 18 10 6 1
Censored 0 0 0 0 7 11 16
Events 0 8 13 14 15 15 15
TNFAIP8 low
At risk 32 25 22 20 9 2 0
Censored 0 0 0 0 11 17 19
Events 0 7 10 12 12 13 13

Figure S12. Kaplan Meier curves depicting the survival in high and low groups of the DNA methylation markers, PSA and PSMA-TV levels using their respective median values of the CRPC cohort as stratifying cut-off.

References

1. Scher HI, Morris MJ, Stadler WM, Higano C, Basch E, Fizazi K, et al. Trial Design and Objectives for Castration-Resistant Prostate Cancer: Updated Recommendations From the Prostate Cancer Clinical Trials Working Group 3. J Clin Oncol. 2016;34:1402–18.

2. Dillinger T, Sheibani-Tezerji R, Pulverer W, Stelzer I, Hassler MR, Scheibelreiter J, et al. Identification of tumor tissue-derived DNA methylation biomarkers for the detection and therapy response evaluation of metastatic castration resistant prostate cancer in liquid biopsies. Mol Cancer. 2022;21:7.

3. Beikircher G, Pulverer W, Hofner M, Noehammer C, Weinhaeusel A. Multiplexed and Sensitive DNA Methylation Testing Using Methylation-Sensitive Restriction Enzymes "MSRE-qPCR." Methods Mol Biol. 2018;1708:407–24.

4. Seifert R, Emmett L, Rowe SP, Herrmann K, Hadaschik B, Calais J, et al. Second version of the prostate cancer molecular imaging standardized evaluation framework including response evaluation for clinical trials (PROMISE V2). Eur Urol. 2023;83:405–12.
